# Genome-Wide DNA Methylation Analysis in Male Methamphetamine Users With Different Addiction Qualities

**DOI:** 10.3389/fpsyt.2020.588229

**Published:** 2020-10-23

**Authors:** Liang Liu, Tao Luo, Huixi Dong, Chenxi Zhang, Tieqiao Liu, Xiangyang Zhang, Wei Hao

**Affiliations:** ^1^Department of Geriatric Psychiatry, Wuxi Mental Health Center, Nanjing Medical University, Wuxi, China; ^2^Hunan Key Laboratory of Psychiatry and Mental Health, Department of Psychiatry and Mental Health Institute of the Second Xiangya Hospital, National Clinical Research Center on Mental Disorders, National Technology Institute on Mental Disorders, Central South University, Changsha, China; ^3^Department of Clinic Psychiatry, Jiangxi Mental Hospital, Nanchang University, Nanchang, China; ^4^Institute of Psychology, Chinese Academy of Sciences, Beijing, China

**Keywords:** methamphetamine, addiction quality, genome-wide DNA methylation analysis, circadian entrainment pathway, caveolin-2

## Abstract

This paper aimed to explore the genome-wide DNA methylation status of methamphetamine (MA) abusers with different qualities to addiction and to identify differentially methylated candidate genes. A total of 207 male MA abusers with an MA abuse frequency of ≥10 times and an MA abuse duration of ≥1 year were assigned to the high MA addiction quality group (HMAQ group; 168 subjects who met the diagnostic criteria for MA dependence according to the DSM-IV) or to the low MA addictive quality group (LMAQ group; 39 subjects who did not meet the criteria for MA dependence). In addition 105 healthy controls were recruited. Eight HMAQ subjects, eight LMAQ subjects, and eight healthy controls underwent genome-wide DNA methylation scans with an Infinium Human Methylation 450 array (Illumina). The differentially methylated region (DMR) data were entered into pathway analysis, and the differentially methylated position (DMP) data were screened for candidate genes and verified by MethyLight qPCR with all samples. Seven specific pathways with an abnormal methylation status were identified, including the circadian entrainment, cholinergic synapse, glutamatergic synapse, retrograde endocannabinoid signaling, GABAergic synapse, morphine addiction and PI3K-Akt signaling pathways. SLC1A6, BHLHB9, LYNX1, CAV2, and PCSK9 showed differences in their methylation levels in the three groups. Only the number of methylated copies of CAV2 was significantly higher in the LMAQ group than in the HMAQ group. Our findings suggest that the circadian entrainment pathway and the caveolin-2 gene may play key roles in MA addiction quality. Further studies on their functions and mechanisms will help us to better understand the pathogenesis of MA addiction and to explore new targets for drug intervention.

## Introduction

Methamphetamine (MA) is an amphetamine-type stimulant drug with a strong propensity for abuse, and it promotes the release of monoamine neurotransmitters in the central and peripheral systems (1). A low dose of MA can induce a series of short-term effects, including euphoria, an increase in energy, positive emotions, increases in heart rate and blood pressure, mydriasis, peripheral high fever, a loss of appetite and weight, improvements in mental acuity, and social and sexual disinhibition (2). A high dose or repeated abuse of MA can cause mental disorders with typical symptoms, including paranoid delusions, auditory hallucinations, increased activity, and stereotypic behavior (3).

To date, MA abuse remains a serious social problem worldwide. The report of the United Nations Office on Drugs and Crime (UNODC) shows that amphetamine-type stimulants, especially MA, rank as the second most widely used illegal drugs worldwide, and 0.7% of the world's population used amphetamine-type stimulants in 2016 (4). According to the 2017 Report on Drug Control in China, by the end of 2017, more than 1.54 million individuals abused synthetic drugs (especially MA), accounting for 60.2% of drug abusers, which did not include double hidden drug users (5). Numerous clinical observations have indicated that different individuals experiencing the same level of repeated MA exposure show different qualities to addiction. A large sample epidemiological survey also exhibited the same situation. In 2012, the Substance Abuse and Mental Health Services Administration (SAMHSA) estimated that 12 million individuals (~4.7% of the population aged 12 years or older) had used MA once in their lifetime, 1.2 million individuals (~0.4% of the population aged 12 years or older) had used MA in the past year, and 0.44 million individuals (~0.2% of the population aged 12 years or older) had used MA in the past month (6). Moreover, 0.54 million individuals (also ~0.2% of the population aged 12 years or older, roughly close to the proportion of the individuals using MA in the past month) in the United States in 2012 met the diagnostic criteria for stimulant drug abuse or dependence according to the DSM-IV (6).

However, the mechanisms for differences in quality to addiction remain unclear. Over the years, classic DNA polymorphism studies have not clearly resolved this issue. Most of the studies have focused on the potential susceptible genetic variations for addiction and have found that genetic polymorphisms of multiple candidate genes are significantly associated with susceptibility to addiction (7). It should be noted, however, that the currently identified risk locus associated with addiction susceptibility could account for only a moderate degree of the genetic variance (<5%) (8). Moreover, the results of molecular genetic studies are non-repeatable because of the small sample size and significant heterogeneity. In general, differences in quality to addiction among different individuals are currently thought to arise from genetic predisposition and the environment (e.g., acute drug exposure). At present, epigenetic changes are generally considered to be manifestations of genetic-environment interactions. Epigenetic refers to heritable but reversible regulation of various genetic functions, including gene expression mediated by modifications of DNA and histones (9). Various aspects of epigenetic changes are very stable, making them ideal vectors for addiction susceptibility research (10).

Disturbances in epigenetics, particularly DNA methylation, have been implicated in the pathophysiology of several psychiatric disorders, such as eating disorders (11), depression (12), schizophrenia (13), cocaine addiction (14), and alcohol dependence (15). Epigenetic research on MA addiction is at a relatively early stage, far behind that of cancer or other complex disorders, and far behind that of cocaine, heroin, and other substance abuse. A review discussed recent advances in epigenetic mechanisms underlying amphetamine- or MA-induced behavioral, transcriptional, and synaptic plasticity, and found drug exposure induces major epigenetic modifications in a very complex manner, such as histone acetylation and methylation, DNA methylation (16). DNA methylation is an important gene regulatory mechanism underlying MA-induced learning and memory alterations (17). The genome-wide epigenetic approaches will accelerate the speed of discovery in the field of addiction. However, there has only a few genome-wide DNA methylation study on MA. Desplats et al. (18) reported genome-wide profiling of DNA methylation in postmortem frontal cortex tissue of with HIV-1 infection and MA dependence patients showed differential methylation on genes related to neurodegeneration; dopamine metabolism and transport; and oxidative phosphorylation. Cadet et al. (19) reported genome-wide DNA hydroxymethylation analysis of rats with MA exposure indicated that changes in differentially hydroxymethylated regions and increased expression of specific potassium channels in the NAc may promote abstinence from drug-taking behaviors. González et al. (20) reported that the global DNA cytosine methylation (5-mC) levels in genomic DNA were significantly increased in the medial prefrontal cortex (mPFC) of mice treated with a 7-days repeated MA administration.

This study aimed to explore the genome-wide DNA methylation status of individuals with different qualities to MA abuse through a two-step experiment, including screening for candidate genes with genome-wide DNA methylation microarrays and verifying abnormally methylated genes from a large number of samples using MethyLight quantitative polymerase chain reaction, which were divided into samples from high MA addictive quality and low MA addictive quality groups.

## Materials and Methods

### Participants

Two hundred and seven male MA abusers, with an MA abuse frequency of ≥10 times and an MA abuse duration of ≥1 year from 577 volunteer participants of Xinkaipu Compulsory Rehabilitation Institute (Changsha, Hunan) and White Mud Lake Compulsory Rehabilitation Institute (Xiangyin, Hunan), underwent a Structured Clinical Interview for DSM-IV Axis I Disorders (SCID-I/P) from March 2013 to January 2014. Compulsory rehabilitation is the primary form of treatment for illegal drug dependence (61.6% drug users received compulsory treatment by 2012) in China (21). The residential MA users were admitted in the treatment facilities after confirmed MA use by urine or hair test. Among them, 168 individuals were classified into the high MA addictive quality group (HMAQ group; MA-dependent patients according to the DSM-IV), and 39 individuals were classified into the low MA addictive quality group (LMAQ group; did not meet the diagnostic criteria for MA dependence). In addition, 105 healthy controls who received physical examinations at the Second Xiangya Hospital were recruited.

A complete medical history, physical examination and laboratory tests were obtained from all subjects. All of them were male, Han Chinese, aged 18–50 years, and without severe acute or chronic medical illnesses. The demographic data and substance abuse characteristics of the three groups are shown in Table 1. Polydrug abuse in methamphetamine users is very common (22), and this paper wants to focus on methamphetamine users. So, the subjects with other illicit drugs use more than three times, including but not limited to heroin, marijuana, and Ketamine, were excluded from this study. There were only 207 male MA abusers met the requirements in 577 volunteer participants from two compulsory rehabilitation institutes. The co-use of alcohol and tobacco were recorded in Table 1.

**Table 1:** Demographic data and substance abuse characteristics of three groups.

	**HMAQ group (*n* = 168)**	**LMAQ group (*n* = 39)**	**Healthy controls (*n* = 105)**
Age (years old)	30.30 ± 6.45	32.21 ± 7.61	30.05 ± 6.51
Usage mode	Heated suction	Heated suction	NA
Total duration of drug abuse (months)^*^	36.78 ± 21.34	26.52 ± 18.10	NA
Age of first drug abuse (years old)	25.31 ± 17.37	28.08 ± 7.52	NA
Withdrawal time (months)	9.75 ± 3.21	10.66 ± 4.48	NA
Relapse number^*^			
1 (first use)	91 (54.17%)	28 (71.79%)	NA
2–3	59 (35.12%)	11 (28.21%)	NA
≥4	18 (10.71%)	0	NA
**Polydrug Abuse History**			
Tobacco	163 (95.83)	36 (92.31)	NA
Alcohol	57 (33.93%)	14 (35.90%)	NA

A total of 207 male MA abusers with MA abuse frequency ≥ 10 times per year and MA abuse time ≥ 1 year were recruited. One hundred and sixty-eight cases were classified to high MA addictive quality group (HMAQ group, MA dependent patients according to DSM-IV), and 39 cases to low MA addictive quality group (LMAQ group) who abused MA at least 10 times and more than 1 year, but did not meet the diagnostic criteria for MA dependence on DSM-IV. Significant differences were noted in the total duration of drug abuse (t = 2.7781, P = 0.006) and relapse numbers (χ^2^ = 6.784, P = 0.032) between the HMAQ and LMAQ groups.

* *p < 0.05*.

This study was conducted in accordance with the principles expressed in the Declaration of Helsinki and was approved by the Ethics Committee of the Second Xiangya Hospital. Prior to the study, the procedure was fully explained, and written informed consent was obtained from each subject. All the subjects were free to participate in or abstain from this study, and free to withdraw from this study anytime without threat of punishment.

### Sample Processing, DNA Extraction, and Bisulfite Conversion

Fasting blood samples were collected and stored at −80°C. Genomic DNA was isolated from blood samples using DNeasy blood & tissue kits (Qiagen, Valencia, CA, USA), and bisulfite modification was performed using the EZ DNA Methylation-Gold kit (Zymo, Irvine, CA) after all blood samples were collected. The bisulfite-converted DNA was resuspended in TE buffer and stored at −80°C until analysis.

### Illumina Infinium® HumanMethylation450 BeadChip Microarray Analysis

We randomly selected eight subjects (dosage: 675.22 ± 93.26 mg/time) in the HMAQ group, eight subjects (dosage: 384.32 ± 87.75 mg/time) in the LMAQ group, and eight subjects in the healthy control group for genome-wide DNA methylation analysis. The bisulfite-converted DNA was then hybridized to the Infinium HumanMethylation450 BeadChip microarray (Illumina, San Diego, USA) in Shanghai Biostar Genechip, Inc. (Shanghai, China) using Illumina-supplied reagents and protocols. This method simultaneously profiles the methylation status for > 485,000 CpG sites at a single-nucleotide resolution. The methylation status of each individual CpG locus was calculated as the ratio of the fluorescent signals (β = Max (M,0)/[Max (M,0) + Max (U,0) + 100]), which ranged from 0 (no methylation) to 1 (complete methylation), using the average probe intensity of the methylated (M) and unmethylated (U) alleles. The quality control information is provided in the supplement as Supplementary Figures 1–3. The original data of the chip were preprocessed using the ChAMP package of R software with the BMIQ method for type 2 probe calibration, the ComBat method of the SVA package for the correction of multiple batch effects, the RefbaseEWAS for cell-type heterogeneity calibration, the minfi package for functional normalization function analysis, and the Lasso method for differentially methylated region (DMR) analysis. The screening of differentially methylated loci screening used the pooled *t*-test with *p* < 0.05 and |beta.difference|>0.14 as the threshold for significant differences.

### Functional Annotation and Pathway Analysis and Potential Candidate Gene Selection

After the genome DNA methylation chip data were analyzed, the differentially methylated positions (DMPs) and differentially methylated regions (DMRs) were screened. DMRs were included in the pathway analysis of the Kyoto Encyclopedia of Genes and Genomes (KEGG, http://www.genome.ad.jp/kegg/), and DMPs were used to screen for candidate genes to perform MethyLight qPCR verification with large samples. To screen for candidate genes from the complex DMP results and to eliminate the impact of confounding factors, the following screening strategies were performed: first, the abnormally methylated sites should correspond to the specific gene name or the UCSC_REFGENE_ACCESSION number; second, the abnormally methylated sites should be located in TSS200 and TSS1500 of the gene promoter region; third, Gene Ontology (GO) analysis (http://www.geneontology.org) should confirm the function of the abnormally methylated genes related to the nervous system; and fourth, the methylation statuses of the candidate gene should be different in all pairwise comparisons of the three groups.

After further literature review and gene functional analysis, solute carrier family 1 member 6 (SLC1A6), basic helix-loop-helix family member b9 (BHLHB9), Ly6/neurotoxin 1 (LYNX1), caveolin 2 (CAV2), and proprotein convertase subtilisin/kexin type 9 (PCSK9) were selected for MethyLight qPCR verification.

### Verification of DNA Methylation With MethyLight qPCR

MethyLight qPCR was used to verify the methylation status of the candidate genes, and it is based on the principle of fluorescence-based real-time PCR with two probes. We prepared standard gene products of SLC1A6, BHLHB9, LYNX1, CAV2, and PCSK9 by the amplification of target DNA fragments and the purification of agarose gel DNA fragments using a Gel Extraction Kit and DNA Methylation Kit (CWBiotech, Beijing, China). Their primers and probes were designed by Beacon Designer 7.0, as shown in Table 2. MethyLight qPCR was performed on a PikoReal 96 Real-Time PCR System (Thermo Fisher Scientific Inc., US) using bisulfite-treated DNA and a GoldStar TaqMan Mixture (CWBiotech, Beijing, China). The MethyLight qPCR standard curves for methylated SLC1A6, BHLHB9, LYNX1, CAV2, and PCSK9 are shown in Supplementary Figures 4–8.

**Table 2:** Primers and probes of the candidate genes for DNA methylation analyses.

**Gene**	**Primers**	**Probes**
SLC1A6	F: 5′-GGAAACAGAGAAGCCTGG-3′ R: 5′-CTCAGGAAGCGCTCATTA-3′	M: 5′-TTCGCAGCCTTCGCCATC-3′ U: 5′-TTCACAGCCTTCGCCATC-3′
BHLHB9	F: 5′- TTAGTGTGGTTTTTTTTAATTTT−3′ R: 5′- TAACATAACAACCACCAC−3′	M: 5′- AACAAACGAACAACTAAAAACCC-3′ U: 5′- AACAAACAAACAACTAAAAACCC−3′
LYNX1	F: 5′- TTAGTTTAGTTAGGTTGGAAA-3′ R: 5′- TTCACATTATCTACACTTCTC-3′	M: 5′- ACCTCGACCTAAACTCAAACCTCAC-3′ U: 5′- ACCTCAACCTAAACTCAAACCTCAC−3′
CAV2	F: 5′- TGAGTGGTTAGTAGGTTAA-3′ R:5′- ACAATCACATCTATAATTATCTTAC-3′	M: 5′- CACGAAAACAAAACCCTAACACAAAACC-3′ U: 5′- CACAAAAACAAAACCCTAACACAAAACC-3′
PCSK9	F: 5′- TTGTGTTTATTATAGAAT-3′ R: 5′- TTTATACTACAAAAATTC-3′	M: 5′- AACGTCATATAAATACATTCAA-3′ U: 5′- AACATCATATAAATACATTCAA-3′

*F, forward; R, reverse; M, Methylated; U, Un-Methylated*.

### Statistical Analysis

The chip data and MethyLight qPCR data were processed by R version 3.2.4 (R Statistics), and raw-intensity files were preprocessed and normalized together in quantiles using the ChAMP package with the BMIQ method for type 2 probe calibration, the ComBat method of the SVA package for the correction of multiple batch effects, the RefbaseEWAS for cell-type heterogeneity calibration, the minfi package for functional normalization function analysis, and the Lasso method for differentially methylated region (DMR) analysis. The screening for differentially methylated loci used the pooled *t*-test with *p* < 0.05 and |beta.difference|>0.14 as the threshold for significant differences. The remaining data were analyzed using SPSS 19.0 software with a *t*-test, chi-squared test, Pearson correlation analysis, and covariance analysis with age as the covariant. The discrete trend of CAV2 methylation level in LMAQ group was very serious after the outliers were winsorized at level 1 and 99% (The sample size of LMAQ group is 39, only the maximum value was assigned as 99%). In covariance analysis, the CAV2 methylation level was adjusted with age as covariance from 1.05E6 ± 9.83E5 to 1.05E6 ± 8.51E4, and its discrete trend was relieved.

## Results

### Analysis of Genome-Wide DNA Methylation Microarray Data in Patients With Different Addictive Qualities

**Figure 1:**
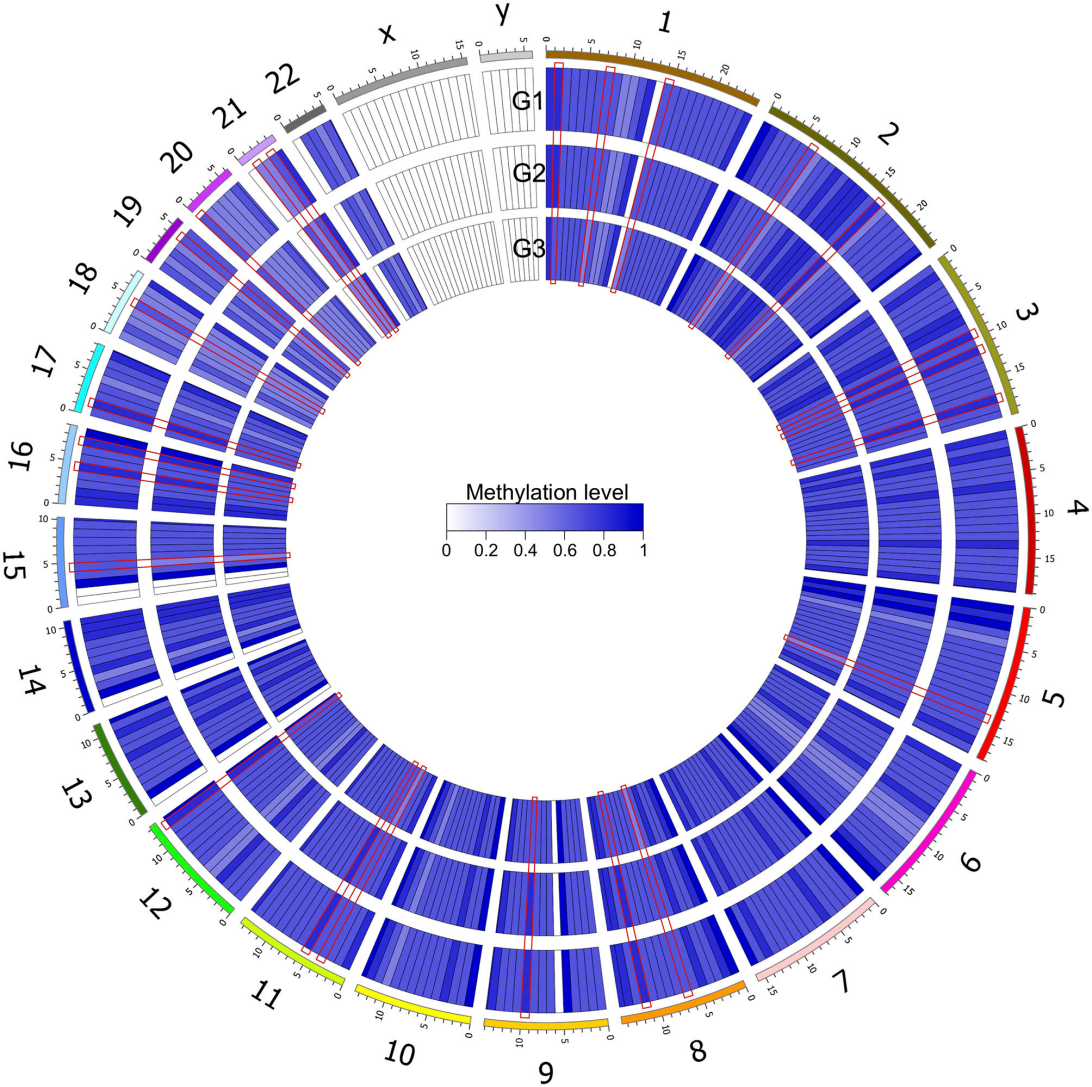
Genome wide methylation level shown in human chromosomes. Taking 10 MB as the sliding window, the average methylation level of each window was calculated. The color depth indicated the average methylation level of the region. G1, HMAQ group; G2, LMAQ group; G3, Health control group.

**Figure 2:**
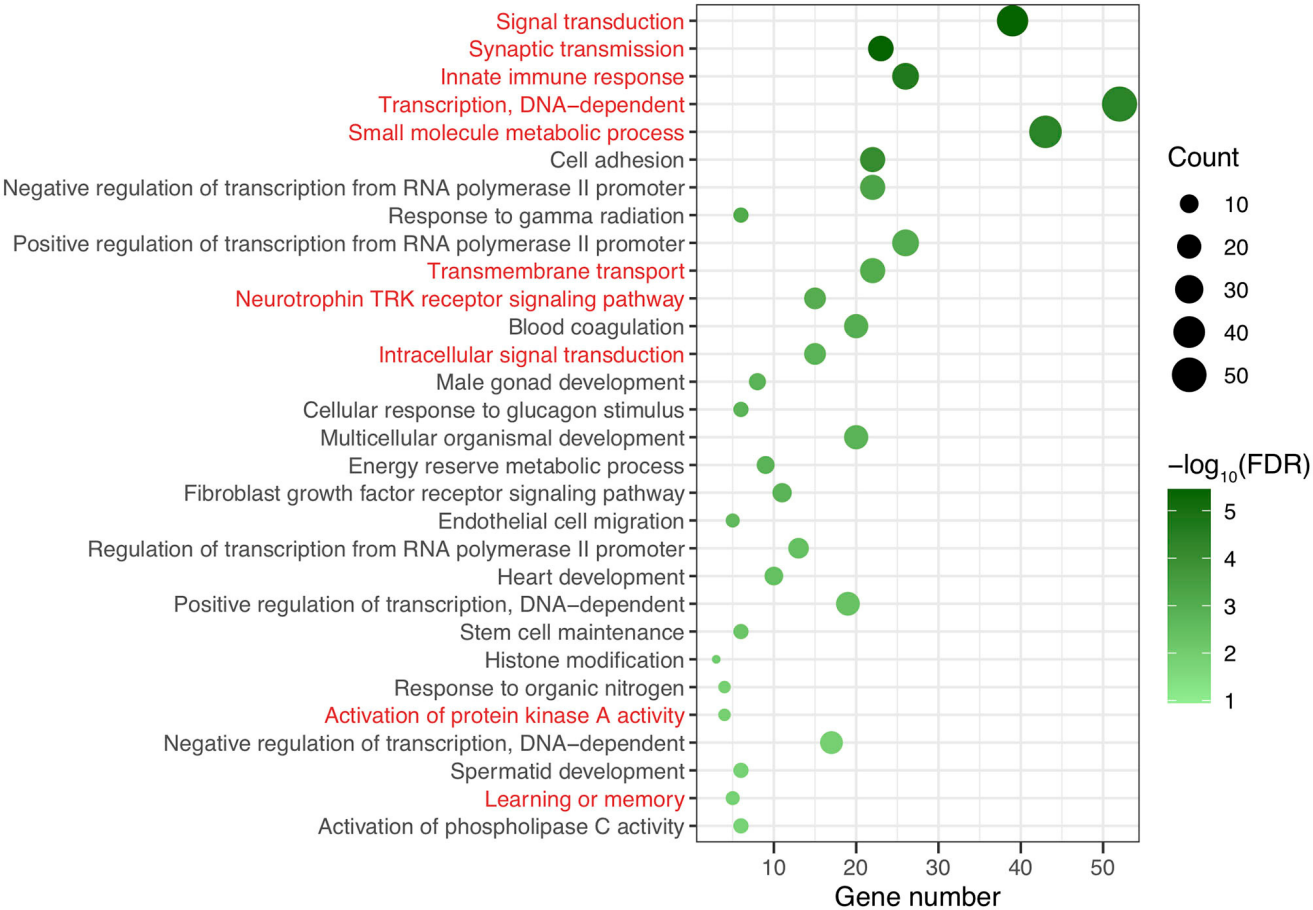
The GO classification analysis results of methylation chip data. The top 30 categories of GO classification analysis results of Methylation chip data were showed. The neural function related classifications with interest of authors were highlighted in red.

**Figure 3:**
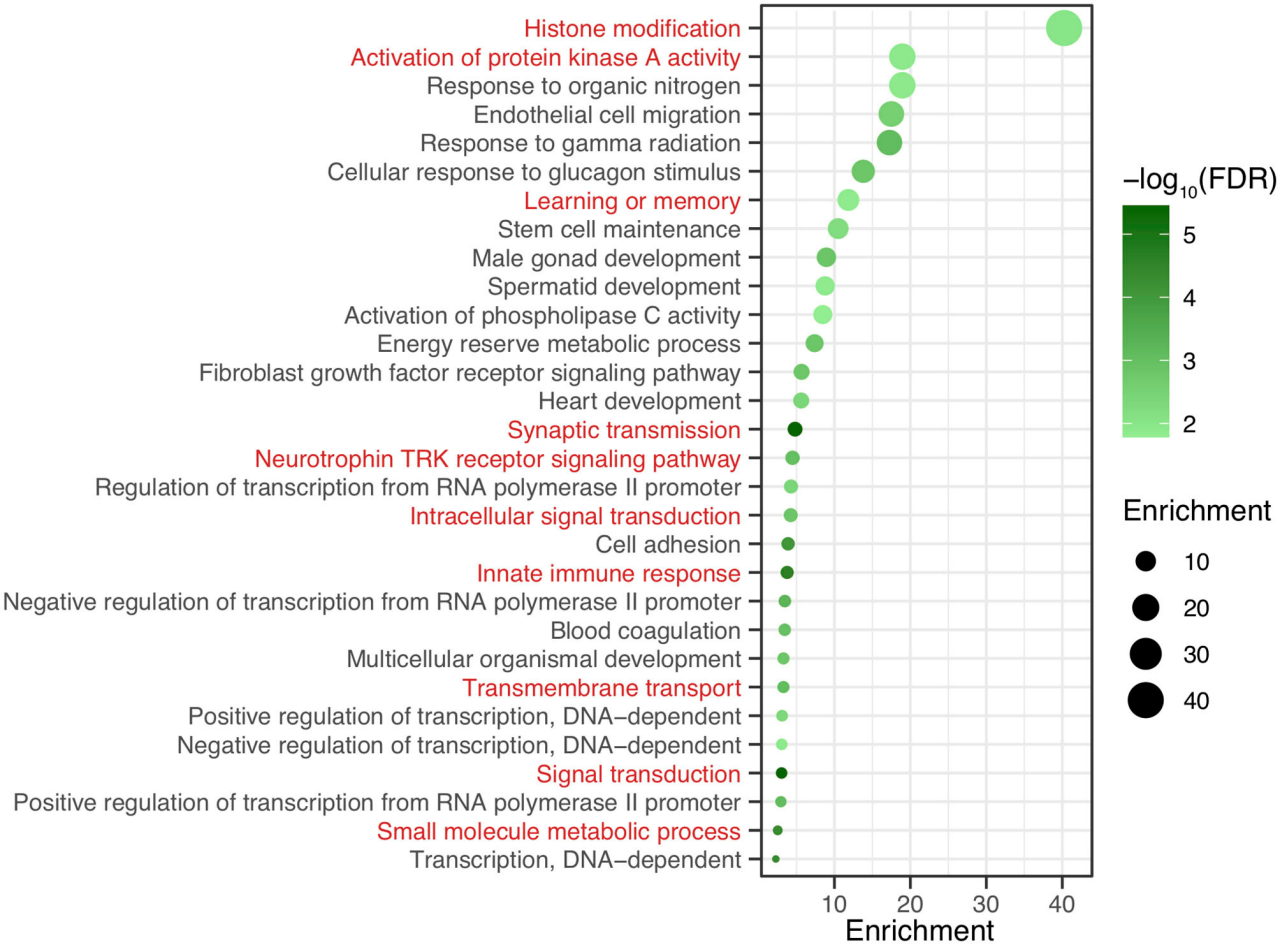
The GO Enrichment analysis results of methylation chip data. The top 30 categories of GO Enrichment analysis results of Methylation chip data were showed. The neural function related enrichments with interest of authors were highlighted in red.

**Figure 4:**
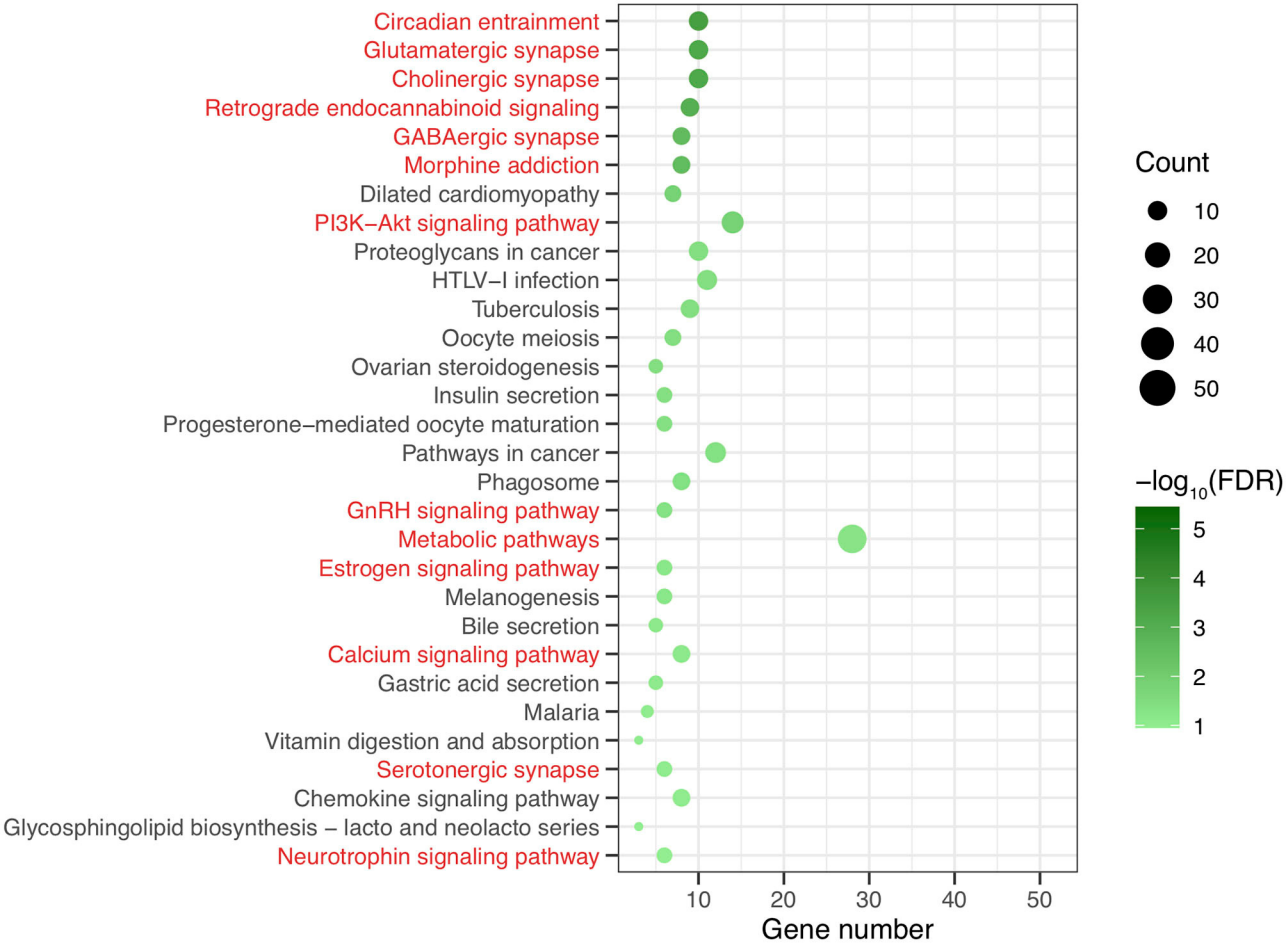
The KEEG pathway analysis results of methylation chip data. The top 30 pathways of KEEG pathway analysis results of Methylation chip data were showed. The neural function related pathways with interest of authors were highlighted in red.

**Figure 5:**
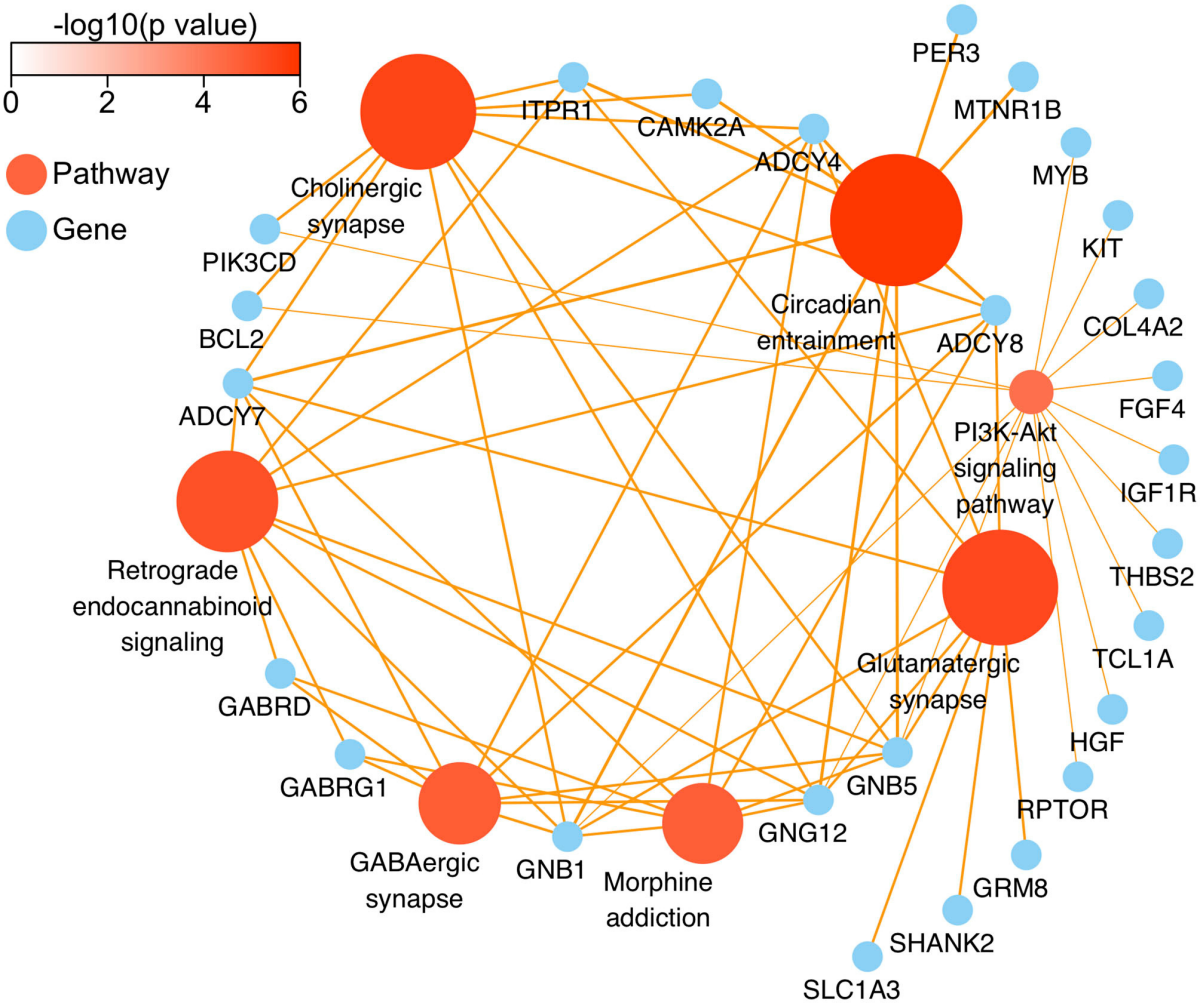
The network diagram of top seven neural specific KEEG pathways of methylation chip data. The neural specific KEEG pathways with the top seven FDR values are shown as brown ball, which inconsistent size is related to their -log_10_(FDR), and the depth of brown is according to -log_10_(p). Their 26 enrichment genes are shown as sky-blue ball. With an orange color, the arrow pointing at the linked pathway, and the inconsistent thickness of the lines are also according to the Enrichment value.

After genomic DNA methylation chip data were analyzed, the genome wide methylation level in human chromosomes was shown in Figure 1. The distribution of differential methylation sites on chromosomes was shown in Supplementary Figure 9. The heatmaps and volcano plots of differential methylation sites in pairwise comparisons of three groups were shown in Supplementary Figures 10, 11. All the chip data had been uploaded in GEO database (GSE154971). After all DMRs in the pairwise comparisons of the three groups were entered into the GO analysis and KEGG pathway analysis, the top 30 categories of the GO analysis and enrichment analysis results of methylation chip data are shown in Figures 2, 3. The top 30 pathways of the KEGG pathway analysis results of methylation chip data are shown in Figure 4. After the non-specific pathways were removed, there were seven pathways associated with the nervous system or neuronal function in the KEGG pathway analysis results of the methylation chip data, as shown in Table 3 and Figure 5.

**Table 3:** The neural specific pathways of genome wide DNA methylation microarray in patients with different addictive qualities.

**Pathway name**	**Diffgene count**	**Gene count**	**Enrichment**	***P*-value**	**FDR**
Circadian entrainment	10	97	8.30	1.54E-06	3.21E-04
Cholinergic synapse	10	113	7.12	6.38E-06	6.57E-04
Glutamatergic synapse	10	118	6.82	9.48E-06	6.57E-04
Retrograde endocannabinoid signaling	9	103	7.03	2.35E-05	1.22E-03
GABAergic synapse	8	90	7.15	6.85E-05	2.85E-03
Morphine addiction	8	93	6.92	8.71E-05	3.02E-03
PI3K-Akt signaling pathway	14	347	3.25	5.31E-04	0.02

Diffgene count: differentially methylated gene count.

*All the DMRs of genome wide DNA methylation microarray in the pairwise comparisons of the three groups were entered into the KEGG pathway analysis, which showed that seven pathways were statistically significant (FDR < 0.05 and p < 0.05) and associated with nervous system or neuronal function*.

**Figure 6:**
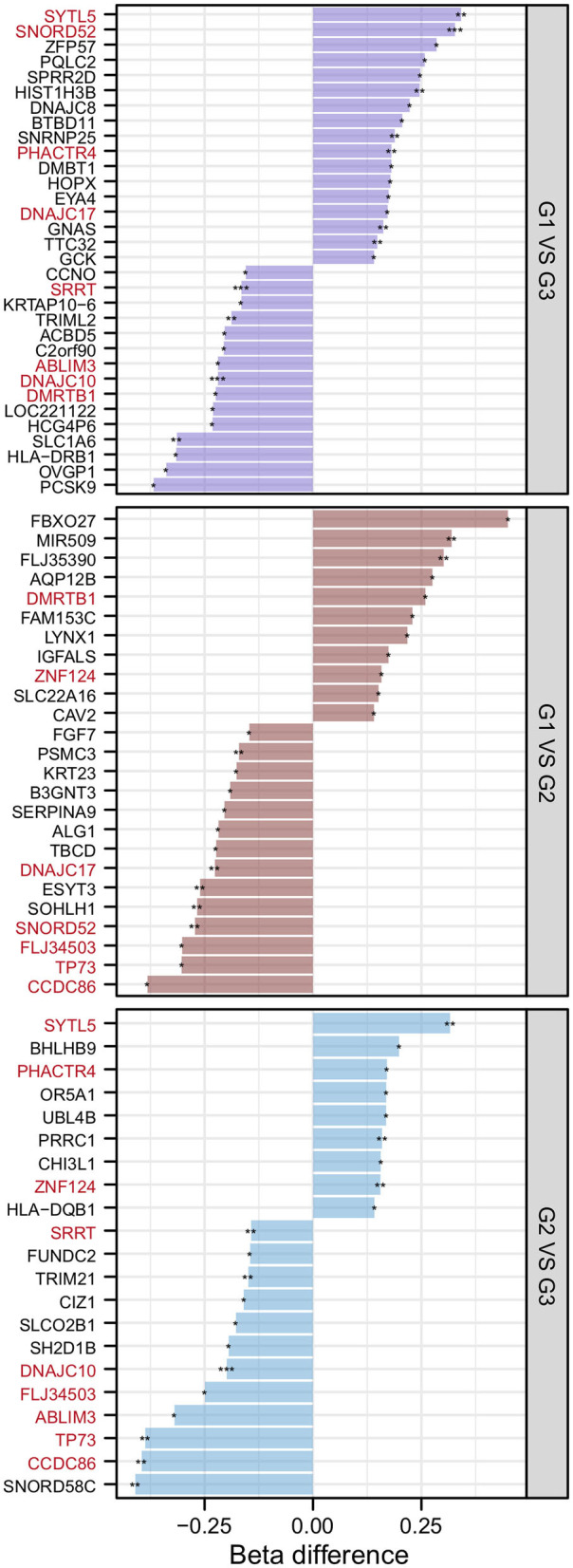
The list of differential DNA methylation genes screened out from methylation chip data. After the DMP results were screened following the strategy described in the Methods section, 78 significantly differentially methylated genes remained. Totaling 32 significantly differentially methylated genes (15 genes with downregulated methylation levels and 17 genes with upregulated methylation levels) between HMAQ patients and healthy controls, 21 significantly differentially methylated genes (12 genes with downregulated methylation levels and nine genes with upregulated methylation levels) between LMAQ patients and healthy controls, and 25 significantly differentially methylated genes (14 genes with downregulated methylation levels and 11 genes with upregulated methylation levels) between HMAQ patients and LMAQ patients.

After the DMP results were screened following the strategy described in the Methods section, 78 significantly differentially methylated genes remained. There were 32 significantly differentially methylated genes (15 genes with downregulated methylation levels and 17 genes with upregulated methylation levels) between HMAQ patients and healthy controls, 21 significantly differentially methylated genes (12 genes with downregulated methylation levels and nine genes with upregulated methylation levels) between LMAQ patients and healthy controls, and 25 significantly differentially methylated genes (14 genes with downregulated methylation levels and 11 genes with upregulated methylation levels) between HMAQ patients and LMAQ patients, as shown in Figure 6.

After further literature review and gene functional analysis, SLC1A6, BHLHB9, LYNX1, CAV2, and PCSK9 were selected for MethyLight qPCR verification with a large sample, and they all had significant differences in the pairwise comparisons of methylation chip data among the HMAQ group, LMAQ group, and healthy control group.

### Comparison of Methylation Levels of SLC1A6, BHLHB9, LYNX1, CAV2, and PCSK9 in the Three Groups

**Figure 7:**
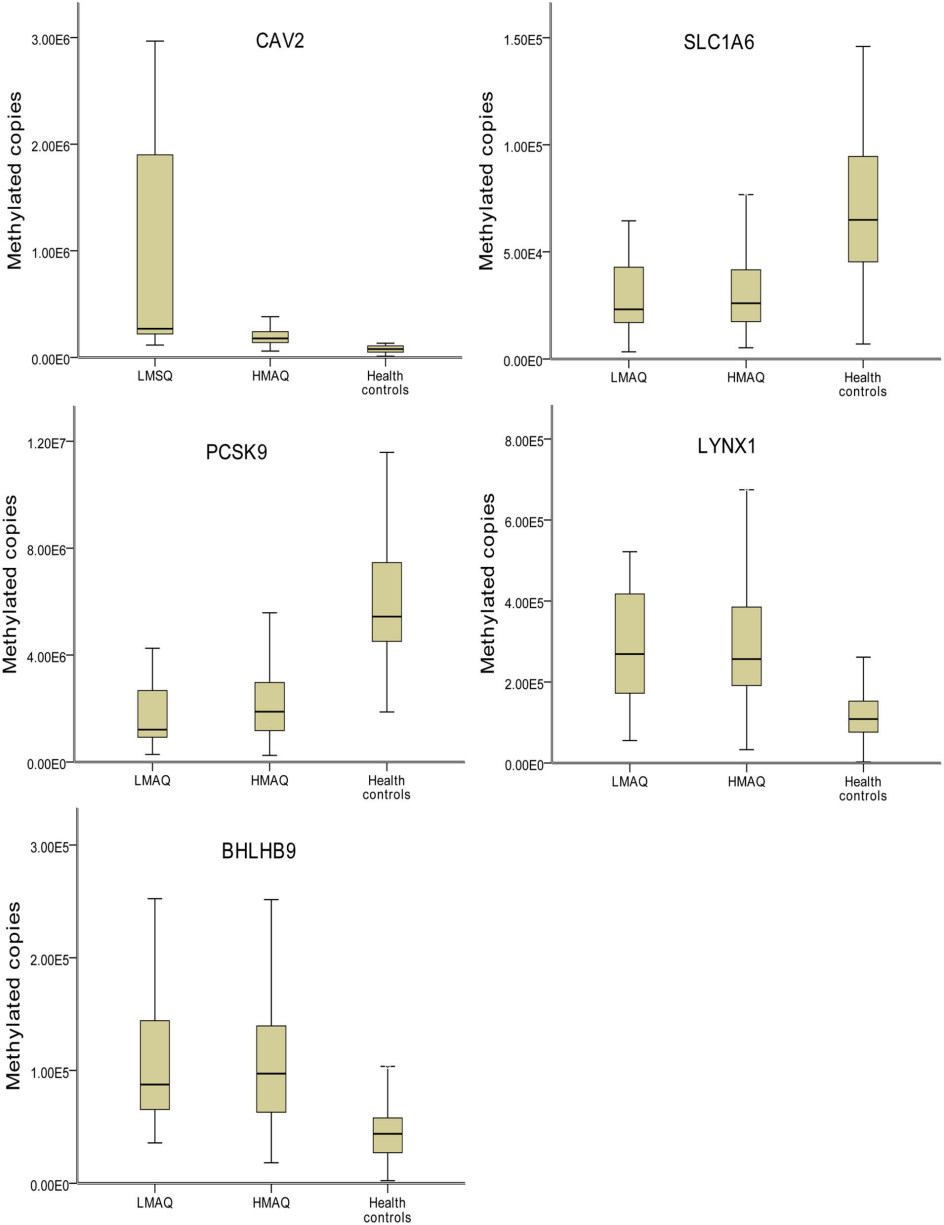
Comparison of methylation levels of SLC1A6, BHLHB9, LYNX1, CAV2, and PCSK9 in three groups. Covariance analysis with age as covariance and Bonferroni *post-hoc* test as pairwise comparisons method showed that the significant differences in the methylated copies of CAV2, BHLHB9, LYNX1, PCSK9, and SLC1A6 in the three groups. The methylation copies of CAV2 were significantly higher in LMAQ than those in HMAQ groups, while the latter were significantly lower than those in healthy control groups. Methylation copies of BHLHB9, LYNX1, PCSK9, and SLC1A6 were not significantly different between the HMAQ and LMAQ groups. However, the methylation copies of BHLHB9 and LYNX1 were significantly higher in both HAMQ and LAMQ than those in healthy control groups, while the methylation copies of PCSK9 and SLC1A6 were significantly lower in both HAMQ and LAMQ than those in the healthy control groups.

MethyLight qPCR verification of DNA methylation results of the five target genes indicated significant differences in the number of methylated copies of CAV2, BHLHB9, LYNX1, PCSK9, and SLC1A6 among the three groups (Table 4 and Figure 7). Pairwise comparisons indicated that the number of methylated copies of CAV2 was significantly higher in the LMAQ group than in the HMAQ group, while it was significantly lower than that in the healthy control group. The number of methylated copies of BHLHB9, LYNX1, PCSK9, and SLC1A6 was not significantly different between the HMAQ group and the LMAQ group. However, the number of methylated copies of BHLHB9 and LYNX1 was significantly higher in both the HMAQ group and the LMAQ group than in healthy control group, while the number of methylated copies of PCSK9 and SLC1A6 was significantly lower in both the HMAQ group and the LMAQ group than in the healthy control group.

**Table 4:** Comparison of methylation copy numbers of the candidate genes in three groups (Unadjusted: ± *SD*; Adjusted: ± *SE*).

	**HMAQ group (*****n*** **=** **168)**		**LMAQ group (*****n*** **=** **39)**		**Health control group (*****n*** **=** **105)**		***F***	***p***
	**Unadjusted**	**Adjusted**	**Unadjusted**	**Adjusted**	**Unadjusted**	**Adjusted**		
CAV2^a^^,^ b^,^ ^c^	3.43E5 ± 5.08E5	3.45E5 ± 4.10E4	1.05E6 ± 9.83E5	1.05E6 ± 8.51E4	1.78E5 ± 2.65E5	1.77E5 ± 5.18E4	38.673	0.000
BHLHB9^b^^,^ ^c^	1.09E5 ± 6.42E4	1.09E5 ± 4.34E3	1.18E5 ± 7.39E4	1.18E5 ± 9.01E3	4.56E4 ± 2.69E4	4.56E4 ± 5.49E3	47.075	0.000
PCSK9^b^^,^ ^c^	2.39E6 ± 1.62E6	2.40E6 ± 1.41E5	1.85E6 ± 1.40E6	1.84E6 ± 2.93E5	5.89E6 ± 2.23E6	5.89E6 ± 1.78E5	136.373	0.000
SLC1A6^b^^,^ ^c^	3.11E4 ± 1.86E4	3.10E4 ± 2.01E3	3.03E4 ± 2.06E4	3.05E4 ± 4.18E3	7.05E4 ± 3.61E4	7.06E4 ± 2.55E3	80.477	0.000
LYNX1^b^^,^ ^c^	3.01E5 ± 1.59E5	3.01E5 ± 1.15E4	3.27E5 ± 2.33E5	3.26E5 ± 2.38E4	1.18E5 ± 6.85E4	1.18E5 ± 1.45E4	56.107	0.000

HMAQ group (high MA addictive quality group): MA abusers conformed to the diagnostic criteria of MA dependence according to the SCID-I/P with drug abuse frequency≥10 times and drug abuse time≥1 year; LMAQ group (low MA addictive quality group): MA abusers did not conform to the diagnostic criteria of MA dependence according to the SCID-I/P with drug abuse frequency≥10 times and drug abuse time≥1 year.

No linear regression between the interaction of age * group and the methylation level of SLC1A6 (F = 0.637, p = 0.530), CAV2 (F = 1.971, p = 0.141), LYNX1 (F = 0.004, p = 0.996), PCSK9 (F = 1.184, p = 0.308), BHLHB9 (F = 0.534, p = 0.587), respectively were found. Covariance analysis with age as covariance was used to compare the methylation copy numbers of the candidate genes in three groups, and Bonferroni post-hoc test was used to do the pairwise comparisons.

a The pairwise comparison between HMAQ group and LMAQ group, p < 0.05.

b The pairwise comparison between HMAQ group and health control group, p < 0.05.

c *The pairwise comparison between LMAQ group and health control group, p < 0.05*.

### Correlation Analysis of Candidate Gene Methylation Levels With MA Abuse Characteristics in the Three Groups

The number of methylated copies of the CAV2 gene was significantly associated with age and the total duration of drug abuse in the LMAQ group. The number of methylated copies of the BHLHB9 gene was significantly correlated with the age of first drug abuse and the total duration of drug abuse in the LMAQ group. No other correlation was identified in the HMAQ group or the healthy control group (Table 5).

**Table 5:** Correlation analysis of candidate gene methylation levels with methamphetamine abuse characteristics in three groups (*r*).

	**HMAQ group (*****n*** **=** **168)**			**LMAQ group (*****n*** **=** **39)**			**Health control (*n* = 105)**
	**Age**	**Age of first drug abuse**	**Total drug abuse time (months)**	**Age**	**Age of first drug abuse**	**Total drug abuse time (months)**	**Age**
CAV2	−0.101	0.105	−0.069	0.351^*^	0.308	0.405^*^	0.078
BHLHB9	0.103	0.129	0.145	0.288	0.368^*^	0.312^*^	−0.132
PCSK9	−0.014	0.004	0.156	−0.235	−0.191	0.134	0.046
SLC1A6	0.146	−0.098	0.009	0.117	0.129	−0.059	0.017
LYNX1	0.023	−0.044	0.104	−0.149	−0.057	−0.203	−0.129

Methylation copies of the CAV2 gene were significantly associated with age and total duration of drug abuse in the LMAQ group. The methylated copies of the BHLHB9 gene were significantly correlated with the age of first drug abuse and the total duration of drug abuse in the LMAQ group.

* *p < 0.05*.

## Discussion

To the best of our knowledge, this is the first study to examine genome-wide DNA methylation in patients who abuse MA. The main findings of this study included (1) the important role of the circadian entrainment pathway in the pathogenesis of MA addiction inferred by the analysis of DMR data and (2) the important role of CAV2 in different qualities mechanisms of MA addiction revealed by DMP data.

### Analysis of DMR Data Inferred the Role of the Circadian Entrainment Pathway in the Pathogenesis of MA Addiction

In this study, all DMRs of the genomic DNA methylation chip data in the pairwise comparisons of the three groups were included in the KEGG pathway analysis, which showed that seven specific pathways were statistically significant. Moreover, all seven pathways, including the Circadian entrainment (23), Cholinergic synapse (24), Glutamatergic synapse (25), Retrograde endocannabinoid signaling (26), GABAergic synapse (27), Morphine addiction (28), and PI3K-Akt signaling pathways (29), were identified to be associated with MA addiction or reward circuits.

The most significant finding of our current study was that the circadian entrainment pathway, which plays an important role in helping organisms cope with dynamic environmental changes and maintain survival with adaptive behavior, was involved in susceptibility to MA addiction (30). In mammals, the circadian entrainment pathway consists of two parts. One is a light signal mechanism that converts light signals into glutamic acid and pituitary adenylate cyclase activating peptide (PACAP) signals and stimulates the suprachiasmatic nucleus (SCN) of the hypothalamus to form a circadian rhythm. Then, the SCN connects the auxiliary oscillators of the whole brain and the body through peptide and neurotransmitter signals (including dopamine, glutamic acid, gamma-aminobutyric acid, etc.) and regulates the physiology and behavior of organisms in a 24-h cycle with molecular clocks throughout various cells throughout the body (31). Second, in the non-optical signaling mechanism, melatonin inhibits light-induced circadian rhythms by binding to melatonin receptors (MT1, MT2) to inhibit adenylate cyclase (AC) (32).

The circadian rhythm transcription factors are also deeply involved in the mechanism of the reward pathway. The SCN directly sends gamma-aminobutyric acid (GABA)-ergic projections to connect the lateral habenular nucleus (LHb), dorsomedial hypothalamic nucleus (DmH), medial anterior optic area (mPOA) and paraventricular thalamic nucleus (PVT), which can affect reward circuits through indirect neural connections (33). The crucial brain structural regions in the reward circuits, such as the ventral tegmental area (VTA), nucleus accumbens (NAc), prefrontal cortex (PFC), hippocampus (Hipp) and amygdala (Amy), have been shown to express proteins related to clock regulation (circadian transcription factors) (34) and non-clock regulation (melatonin receptors) (32) to maintain circadian rhythm stability and control emotion and reward behavior through neural circuits and molecular mechanisms (35).

Further, circadian rhythm transcription factors play an important role in the pathogenesis of substance addiction disorder. First, genome-wide association studies (GWASs) have shown close relationships between polymorphisms and other mutations in the circadian rhythm core genes and the pathogenesis of addiction disorder (35). Second, circadian rhythm disorders increase susceptibility to addiction. Repeated light/dark conversion can increase alcohol intake (36) and the preference for MA (37) in pre-exposure-treated rats. In humans, epidemiological studies reported that shift work or jet lag was associated with increased smoking and alcohol consumption (38, 39). Third, substance abuse can alter the functional state of the circadian entrainment pathway. Acute or chronic substance abuse in rodents has been found to alter the expression of circadian rhythm genes, including per1, per3, cry1, bmal1, npas2, and clock, in brain areas such as the NAc, dorsal striatum and hippocampus (40). Finally, manipulation of circadian rhythm genes can significantly alter dopaminergic signaling and reward-related behavior. ClockΔ19 mice showed a high dopaminergic state characterized by increased tyrosine hydroxylase expression in the VTA region and increased dopamine release in the NAc region, with strong sensitization to cocaine, increased preference for cocaine, increased goal-directed behavior, and increased motivation for the self-administration of cocaine (41).

Another interesting finding is that the results of KEGG pathway analysis inferred the role of the morphine addiction pathway, instead of amphetamine addiction pathway, in the pathogenesis of MA addiction. Georgiou et al. (28) reported chronic methamphetamine use and abstinence can induce brain-region specific neuroadaptations of the m-opioid receptor (MOR) in mice. Regional manipulation of MOR expression in NAc and VTA of mice may be a novel approach to differential modulate MA behavioral sensitization (42). Guo et al. (43) reported acute intragastrical administration of naltrexone, a non-selective opioid receptor antagonist, could significantly reduce the cue-induced drug-seeking behavior of rats induced by intraperitoneal injection of MA after extinction training. Also, we checked the pathway annotation and including genes, and found morphine addiction pathway related to GABAergic synapse pathway and dopaminergic synapse pathway, but amphetamine addiction pathway only related to dopaminergic synapse pathway. Our results also reported other related pathways included cholinergic synapse pathway, glutamatergic synapse pathway, and GABAergic synapse pathway. This situation implies that MA addiction is more complicated than only dopaminergic mechanism.

There are few studies on genome-wide methylation of addictive substances, especially methamphetamine, and they are scattered in brain tissue samples of animal models and human blood samples, so the results are difficult to be inconsistent. Only Desplats et al. (18) reported genome-wide profiling of DNA methylation in postmortem frontal cortex tissue of with HIV-1 infection and MA dependence patients showed differential methylation on genes related to neurodegeneration; dopamine metabolism and transport; and oxidative phosphorylation. Fonteneau et al. (14) reported a genome-wide DNA methylation study with cocaine self-administering rats treated with DNA methyltransferase inhibitor, and the Ingenuity pathway analysis (IPA) of differentially methylated genes from the PFCx showed Akt/PI3K pathway and Rho-GTPase family was involved in the plasticity of axonal growth, synaptogenesis, and spine remodeling, underlying the effect of DNA methyltransferase inhibitor on the observed behavioral changes. Akt/PI3K pathway also highlighted in our results.

Meanwhile, there are a few DNA methylation researches of candidate genes in people or animals addicted to MA. Yuka et al. (44) found that the rate of DNA methylation at six CpG islands of SHATI/NAT8L promoter sites is significantly higher in MA users when compared to healthy subjects. Jayanthi et al. (45) reported that a single prior injection of MA enhanced MA self-administration (SA) and blocked SA-induced changes in DNA methylation and mRNA expression of potassium channels in the rat nucleus accumbens. MA produces a variety of epigenetic effects in the brain, which are seminal to establish long-lasting alterations in neuronal activity. Exposure to high and/or prolonged doses of MA induced persistently demethylation within alpha-synuclein gene (SNCA) promoter, which matches the increase in alpha-synuclein protein. Demethylation was remarkable (10-fold of controls) and steady, even at prolonged time intervals being tested so far (up to 21 days of MA withdrawal) (46). MA-induced changes in long interspersed element-1 (LINE-1) partial methylation patterns are associated with MA-induced paranoia and could explain in part the pathophysiology of this type of psychosis. It is argued that MA-induced neuro-oxidative pathways may have altered LINE-1 partial methylation patterns, which in turn may regulate neuro-oxidative and immune pathways, which may increase risk to develop MA-induced paranoia (47). Veerasakul et al. (48) found that the methylation levels in the parvalbumin (PVALB) promoter were increased in MA-dependent patients using pyrosequencing, which might contribute to the GABAergic deficits associated with MA dependence. Hao et al. (49) reported that the methylation levels of the CHN2 gene, which encodes chimeric protein-2, which regulates axonal pruning, were increased in MA addiction patients. Nohesara et al. (29) demonstrated that DNA hypomethylation of the promoter regions of DRD3, DRD4, MB-COMT, and AKT1 was associated with increased expression of the corresponding genes in MA-dependent patients with psychosis and, to a lesser extent, in non-psychotic MA-dependent patients. Xu et al. (50) measured the methylation levels of five CpGs (CpG1–5) on the BDNF promoter using pyrosequencing, and only the CpG5 methylation level was found to be significantly lower in MA addicts, which played a key role in the regulation of BDNF gene expression. Moreover, significant associations were identified between CpG5 methylation and addiction phenotypes, including tension-anxiety, anger-hostility, fatigue-inertia, and depression-dejection.

### Screening and the Validation of DMP Data Revealed the Role of CAV2 in Different Qualities Mechanisms of MA Addiction

After further literature review and gene functional analysis, SLC1A6, BHLHB9, LYNX1, CAV2, and PCSK9 were selected from the DMP results for MethyLight qPCR verification with a large sample. The results indicated that the number of methylated copies of CAV2 in the LMAQ group was significantly higher than that in the HMAQ group, while the number of methylated copies of CAV2 was significantly lower than that in the HMAQ group and the healthy control group.

The number of methylated copies of BHLHB9, LYNX1, PCSK9, and SLC1A6 was not significantly different between the HMAQ group and the LMAQ group. However, the number of methylated copies of BHLHB9 and LYNX1 was significantly higher in both the HMAQ group and the LMAQ group than in the healthy control group, while the number of methylated copies of PCSK9 and SLC1A6 was lower in both the HMAQ group and the LMAQ group than in the healthy control group.

In the LMAQ group, the number of methylated copies of the CAV2 gene was significantly correlated with age and the total duration of drug abuse. The number of methylated copies of the BHLHB9 gene was significantly correlated with the age of first drug abuse and the total duration of drug abuse in the LMAQ group. No other correlations were identified in the HMAQ group or the healthy control group. These five genes with abnormal methylation levels in MA abusers were all associated with neurological function and neurocognition.

It has been reported that only SLC1A6 and PCSK9 were associated with MA and/or ethanol abuse (51–54). SLC1A6 is also referred to as excitatory amino acid transporter 4 (EAAT4), a high-affinity glutamate transporter. The maintenance of low levels of extracellular glutamate and the avoidance of dose-dependent neurotoxicity can be achieved only by glutamate transporter-mediated cellular uptake, such as SLC1A6, because there is no extracellular glutamate metabolic system in humans (55). Szumlinski et al. (51) identified the hyperglutamatergic state within the nucleus accumbens as a biochemical trait that corresponds to both genetic and idiopathic vulnerability for high MA preferences and consumption. Alshehri et al. (52) found that sequential exposure to ethanol and MA produced an additive effect in the downregulation of glutamate transporter 1 (GLT-1, also EAAT2) in the striatum and hippocampus rather than the glutamate aspartate transporter (GLAST, EAAT1) and cysteine/glutamate antiporter (xCT). Pickering et al. (53) found significantly decreased correct alterations and increased time in the choice area at the Y-maze center, as well as decreased mRNA expression of SLC1A6 in the medial prefrontal cortex of male Wistar rats after 2.5 g/kg ethanol treatment and 5 days of intermittent treatment (53).

BHLHB9, also referred to as p60TRP (transcription regulator protein), is a member of the G-protein-coupled receptor (GPCR)-associated sorting protein (GPRASP) family. BHLHB9 can regulate the survival and differentiation of NGF-dependent neurons, and its expression level is decreased in the brains of Alzheimer's disease (AD) patients (56). BHLHB9, similar to GPRASP1, downregulates the expression of delta opioid receptor 1 (Oprd1) in neural stem cells (57). Mishra et al. demonstrated that BHLHB9 improved memory and learning abilities in BHLHB9 transgenic mice, which was attributed to increased synaptic connections and plasticity (57). Zhao et al. (58) reported that GPCRs interacted with β-site APP cleaving enzyme 1 (BACE1), a key secretase in AD pathogenesis. Moreover, the allosteric modulators and biased ligands of GPCRs displayed potential for the pharmaceutical treatment of AD patients, suggesting the possibility that GPCRs may be a therapeutic target for AD.

LYNX1 is a member of the Ly6/neurotoxin family associated with nicotinic acetylcholine receptors (nAChRs), which affect a wide array of biological processes, including learning and memory, attention, and addiction (59). Lynx1 modulates nAChR function *in vitro* by altering agonist sensitivity and desensitization kinetics. Dopaminergic cells of the substantia nigra pars compacta (SNc) and nearly all of the parvalbumin interneurons (GABAergic neurons) express lynx1 mRNA transcripts, whose dysfunctions are characteristics of psychiatric disorders (60). The titration of the lynx1 dosage can maintain neuronal health, while the lynx1 gene may participate in the trade-off between neuroprotection and augmented learning (61). Lynx1 prevents Aβ1-42-induced cytotoxicity in cortical neurons by competing with binding to nAChR subunits, and cortical Lynx1 levels are decreased in a transgenic mouse model with concomitant β-amyloid and tau pathology, which might have functional and pathophysiological implications in Alzheimer's disease (62).

PCSK9 is a member of the *Bacillus subtilis* protease (proprotein invertase) family, which is involved in the systemic control of blood cholesterol levels, neuronal differentiation, apoptosis, migration and nervous system development (63). Monoclonal antibodies that target PCSK9, a novel lipid-lowering approach, have recently been found to be accompanied by an increase in neurocognitive events (64). Picard et al. (65) found that PCSK9 expression levels in autopsy-confirmed AD brains were elevated in the frontal cortices compared to levels found in controls, both at the mRNA and protein levels. PCSK9 regulation seems to be under tight genetic control in females only, with specific variants that could predispose them to increased risk of AD. Hyperlipidemia regulates neuronal apoptosis in the hippocampal CA3 of mice by increasing PCSK9 and BACE1 expression, which may elucidate the role of lipid metabolism disorder in AD pathogenesis (66). A cross-tissue and cross-phenotypic analysis of genome-wide methylomic variation performed in alcohol use disorder (AUD) using samples from three discovery, four replication, and two translational cohorts indicated that replication in the AUD datasets confirmed PCSK9 hypomethylation. A translational mouse model of AUD showed that alcohol exposure caused a downregulation of PCSK9 (54).

CAV2, a membrane microdomain or “lipid raft,” has emerged as an essential functional module of the cell that is critical for the regulation of nerve growth factor (NGF) signaling and subsequent cell differentiation (67). BDNF increased the level of CAV2 in the rafts of hippocampal neurons for synapse development (68). Single-cell gene expression profiles showed downregulation of CAV2, GNB4, and lipase A in AD Rac1b-positive/p75 (NTR)-labeled basal forebrain (CBF) nucleus basalis neurons (69). CAV2 is also involved in the binding of the dopamine D1 receptor and the positive regulation of the dopamine receptor signaling pathway. Only two studies have reported the relationship between CAV1 and addiction. CAV-1 plays an important role in morphine-induced changes in the structural plasticity of primary cultured mouse cortical neurons (70). The compulsive-like intake behavior of MA was associated with an enhanced expression of D1Rs, increased activity of ERK1/2, and a decrease in Cav-1 expression in the dorsal striatum (71).

There were several limitations in this study. First, the subjects who abused MA in this study were recruited from the rehabilitation facilities in one province of China. They had not used MA for at least 3 months or even 2 years; hence, they were not acute users. Thus, there was a broad range of past frequencies and dosages of MA abuse. Second, in this study, we reported only the genome-wide DNA methylation status of MA abusers with different qualities to addiction and did not further explore its underlying mechanisms. Third, this is a DNA methylation research of peripheral blood cells of MA abusers. There are affirmative differences in the environment and differentiation between peripheral blood cells and neurons, but it is the only convenient method to study the DNA methylation alteration of alive MA abusers. Also, Uno et al. (72) reported that the ratios of Shati/Nat8l CpG island methylation were significantly decreased in both the nucleus accumbens and the peripheral blood of MA-induced murine models of schizophrenia-like phenotype compared with those of control mice. To our knowledge, this is the first study to show similar alterations of DNA methylation status in both the brain and peripheral blood of MA treated mice, which suggests the feasibility of this kind of research. For the next step, gene knockout or transgenic animals should be used to investigate the roles of circadian entrainment pathways and the caveolin-2 gene in the pathogenesis of MA addiction.

## Conclusions

This study investigated the genome-wide DNA methylation status of different addiction qualities in MA abusers and identified seven specific pathways with abnormal methylation status, which were all reported to be associated with MA addiction or reward circuits, especially the circadian entrainment pathway, a relatively new pathogenesis. Moreover, CAV2 was the only gene among the five candidate genes screened from the genome methylation chip data that showed significant differences in methylation levels in all pairwise comparisons of the three groups. Further studies on their functions and mechanisms will help us to better understand the pathogenesis of MA addiction and to explore new targets for drug intervention.

## Data Availability Statement

The datasets generated for this study can be found in GEO database (GSE154971).

## Ethics Statement

The studies involving human participants were reviewed and approved by Ethics Committee of the Second Xiangya Hospital. The participants provided their written informed consent to participate in this study. Written informed consent was obtained from each individual for the publication of any potentially identifiable images or data included in this article.

## Author Contributions

WH was responsible for the study design and supervision. LL, TLu, HD, CZ, and TLi had participated in the acquisition, analysis, and interpretation of the data. LL wrote a draft of the manuscript. XZ critically revised the manuscript for important intellectual content and provided the technical support. All authors contributed to the article and approved the submitted version.

## Conflict of Interest

The authors declare that the research was conducted in the absence of any commercial or financial relationships that could be construed as a potential conflict of interest.
