# Supplementary material for: Genome-Wide DNA Methylation Analysis in Male Methamphetamine Users With Different Addiction Qualities
Source: Front Psychiatry. 2020 Oct 23;11:588229. doi: 10.3389/fpsyt.2020.588229 (PMC7645035; doi:10.3389/fpsyt.2020.588229)
Supplement: Supplementary file 1 [file Data_Sheet_1.doc]

**Supplement**

This supplementary material has been provided by the authors to give readers quality control and other additional information about their work.

**eFigure 1. Sample quality control of Illumina Human Methylation 450K Chip Experiment.** The overall methylation degree of each sample is represented by a dot, and its distribution in the upper right corner of the dotted line indicates that it meets the quality control standard. All the samples in this study meet the quality control standard.

**eFigure 2. The beta value density curve of samples.** The peak values of unmethylated density of all the samples were less than 0.2, and their peak values of methylated density were greater than 0.8. There are only two peak densities per sample (unmethylated peak densities and methylated peak densities). The Beta value of density curve of the samples tended to be consistent. g1: HMAQ group; g2: LMAQ group; g3: Health control group.

**eFigure 3. The Box Plot diagram of normalization Beta value of samples.** This Box Plot diagram is used to represent the overall distribution of the beta values of samples.

**eFigure 4. The Methylight qPCR standard curve of methylated SLC1A6.**

**eFigure 5. The Methylight qPCR standard curve of methylated LYNX1.**

**eFigure 6. The Methylight qPCR standard curve of methylated CAV2.**

**eFigure 7. The Methylight qPCR standard curve of methylated BHLHB9.**

**eFigure 8. The Methylight qPCR standard curve of methylated PCSK9.**

**eFigure 9. The distribution of differential methylation sites on chromosomes.** Taking 10mbp as a window, the number of different methylation sites in the window was counted, and the distribution of sites on chromosome was obtained. (a): The comparison between HMAQ group and LMAQ group; (b): The comparison between HMAQ group and Health control group; (c): The comparison between LMAQ group and Health control group.

**eFigure 10. The heatmap of the differentials of methylation sites in pairwise comparisons of three groups.** The cluster analysis results showed the differentials of methylation sites between groups. Red means high methylation status, and green means low methylation status. The darker color means the more significant methylation differential. g1: HMAQ group; g2: LMAQ group; g3: Health control group.

**eFigure 11. The volcano plot of differential methylation sites in pairwise comparisons of three groups.** In the three volcano plots, the abscissa is delta Beta value, and the ordinate is -logpvalue. The blue rendering points are low methylation sites, and the red rendering sites are high methylation sites. G1: HMAQ group; G2: LMAQ group; G3: Health control group.

**eFigure 1**

**eFigure 1. Sample quality control of Illumina Human Methylation 450K Chip Experiment.** The overall methylation degree of each sample is represented by a dot, and its distribution in the upper right corner of the dotted line indicates that it meets the quality control standard. All the samples in this study meet the quality control standard.

**eFigure 2**


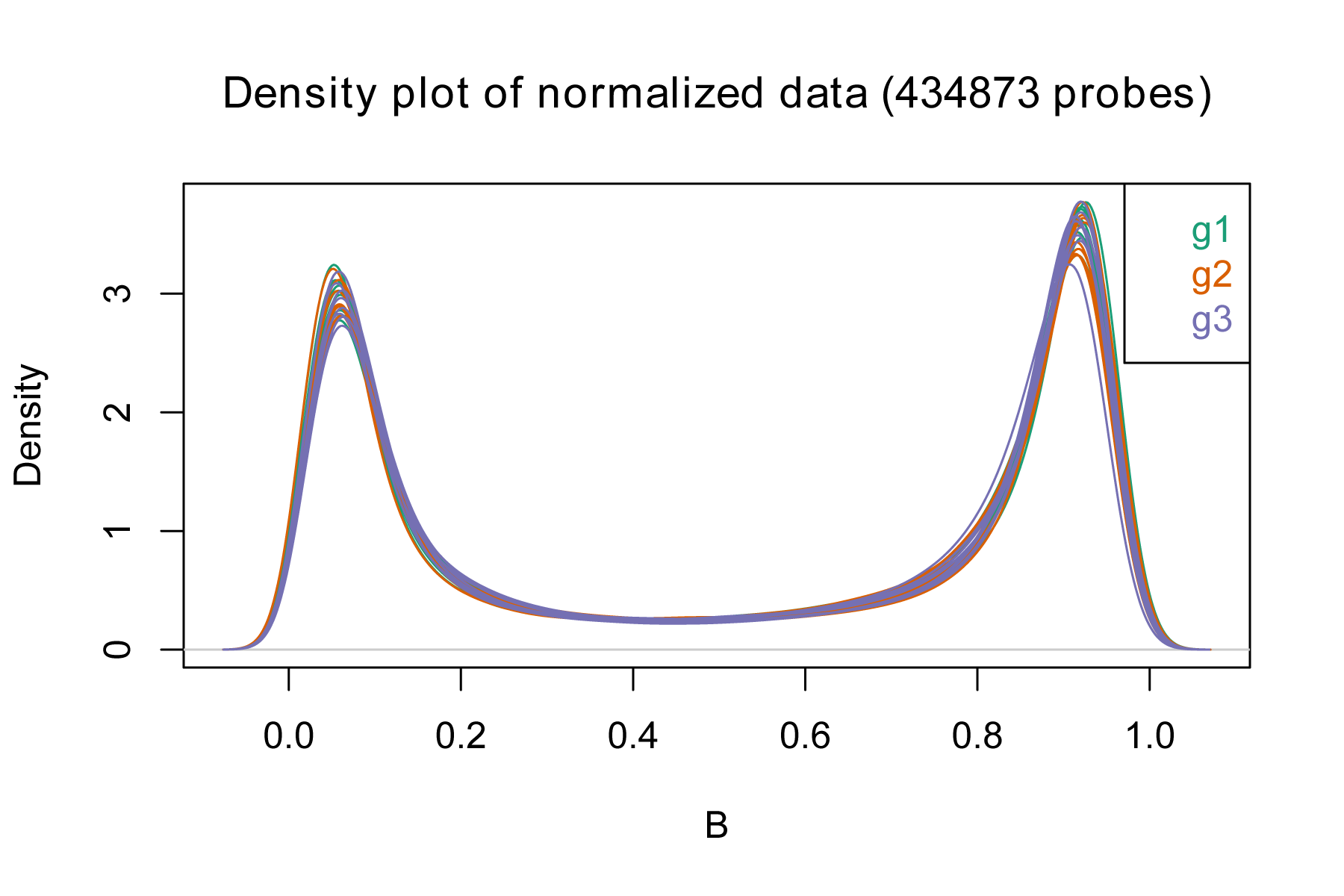


**eFigure 2. The beta value density curve of samples.** The peak values of unmethylated density of all the samples were less than 0.2, and their peak values of methylated density were greater than 0.8. There are only two peak densities per sample (unmethylated peak densities and methylated peak densities). The Beta value of density curve of the samples tended to be consistent. g1: HMAQ group; g2: LMAQ group; g3: Health control group.

**eFigure 3**

**eFigure 3. The Box Plot diagram of normalization Beta value of samples.** This Box Plot diagram is used to represent the overall distribution of the beta values of samples.

**eFigure 4**

**
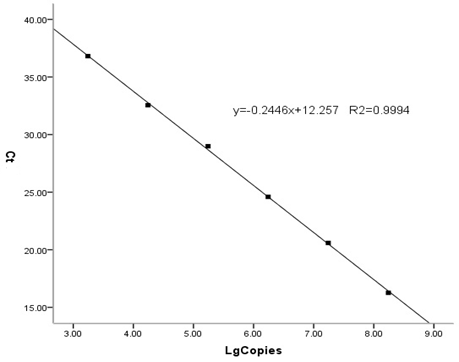
**

**eFigure 4. The Methylight qPCR standard curve of methylated SLC1A6.**


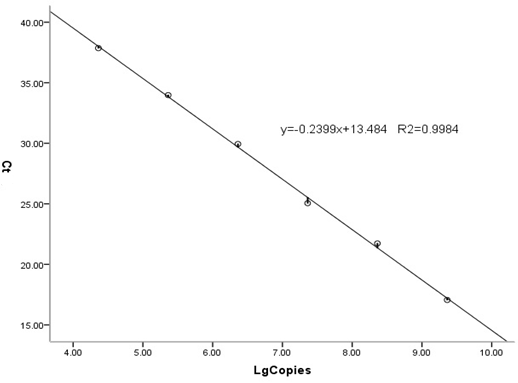
**eFigure 5**

**eFigure 5. The Methylight qPCR standard curve of methylated LYNX1.**

**eFigure 6**


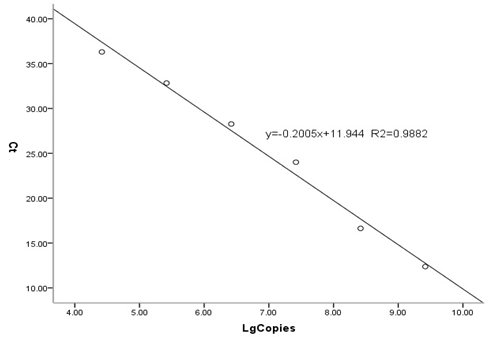


**eFigure 6. The Methylight qPCR standard curve of methylated CAV2.**

**eFigure 7**


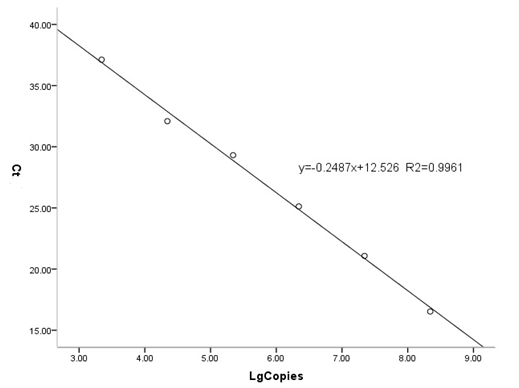


**eFigure 7. The Methylight qPCR standard curve of methylated BHLHB9.**


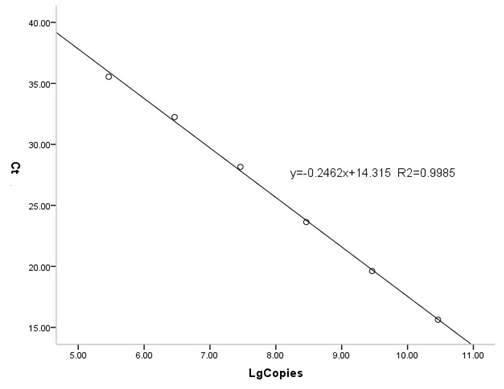
**eFigure 8**

**eFigure 8. The Methylight qPCR standard curve of methylated PCSK9.**

**eFigure 9**

**
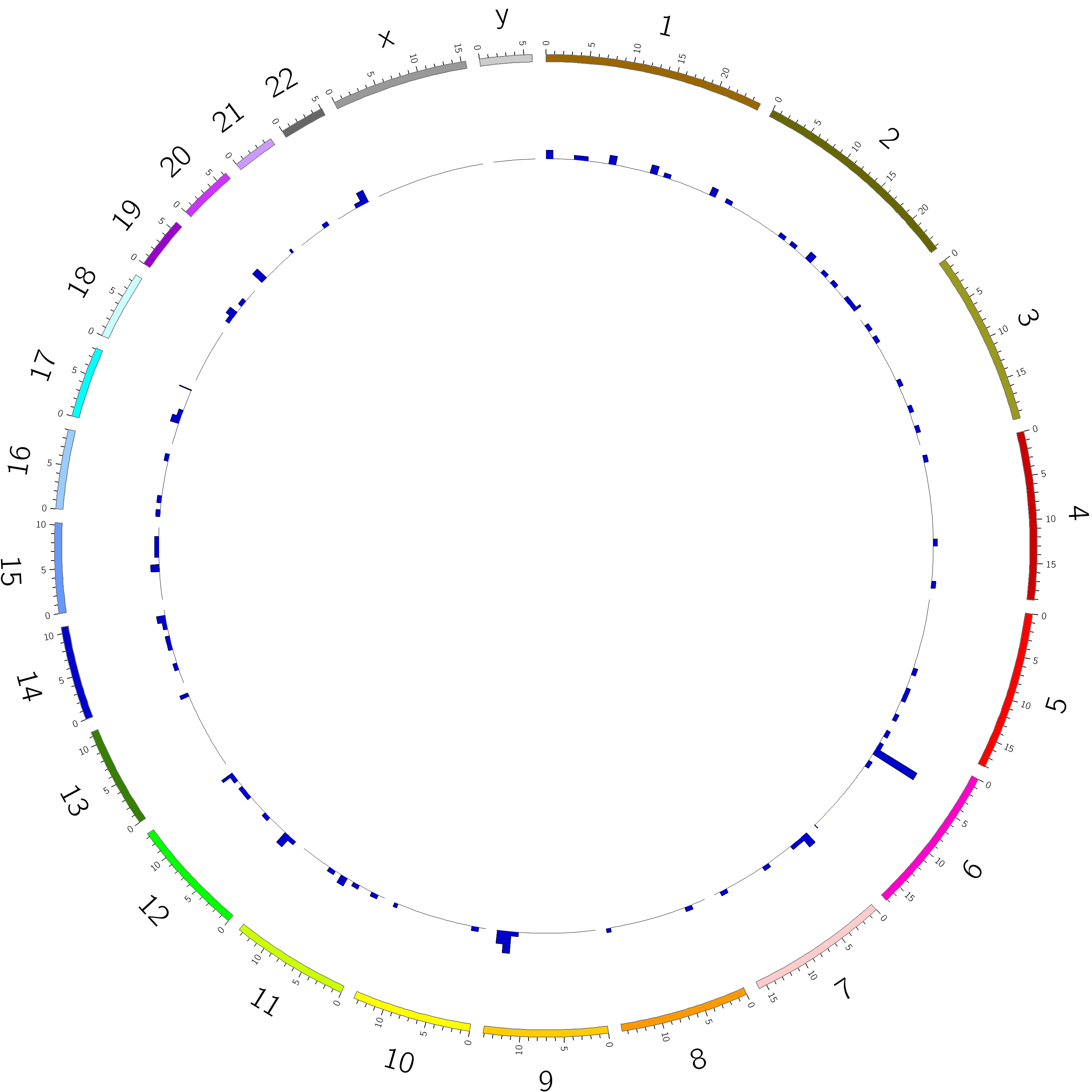
**

**
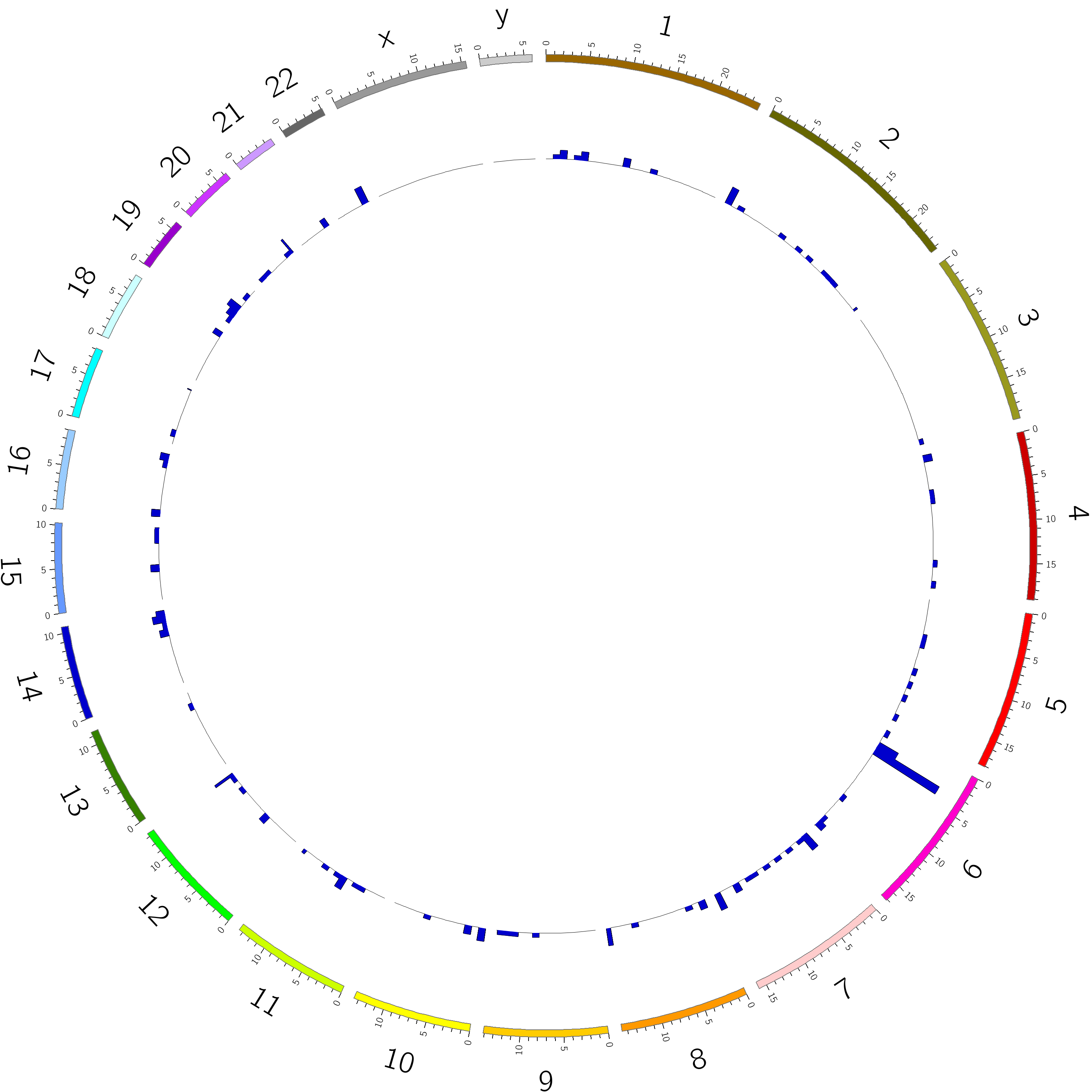
**

**
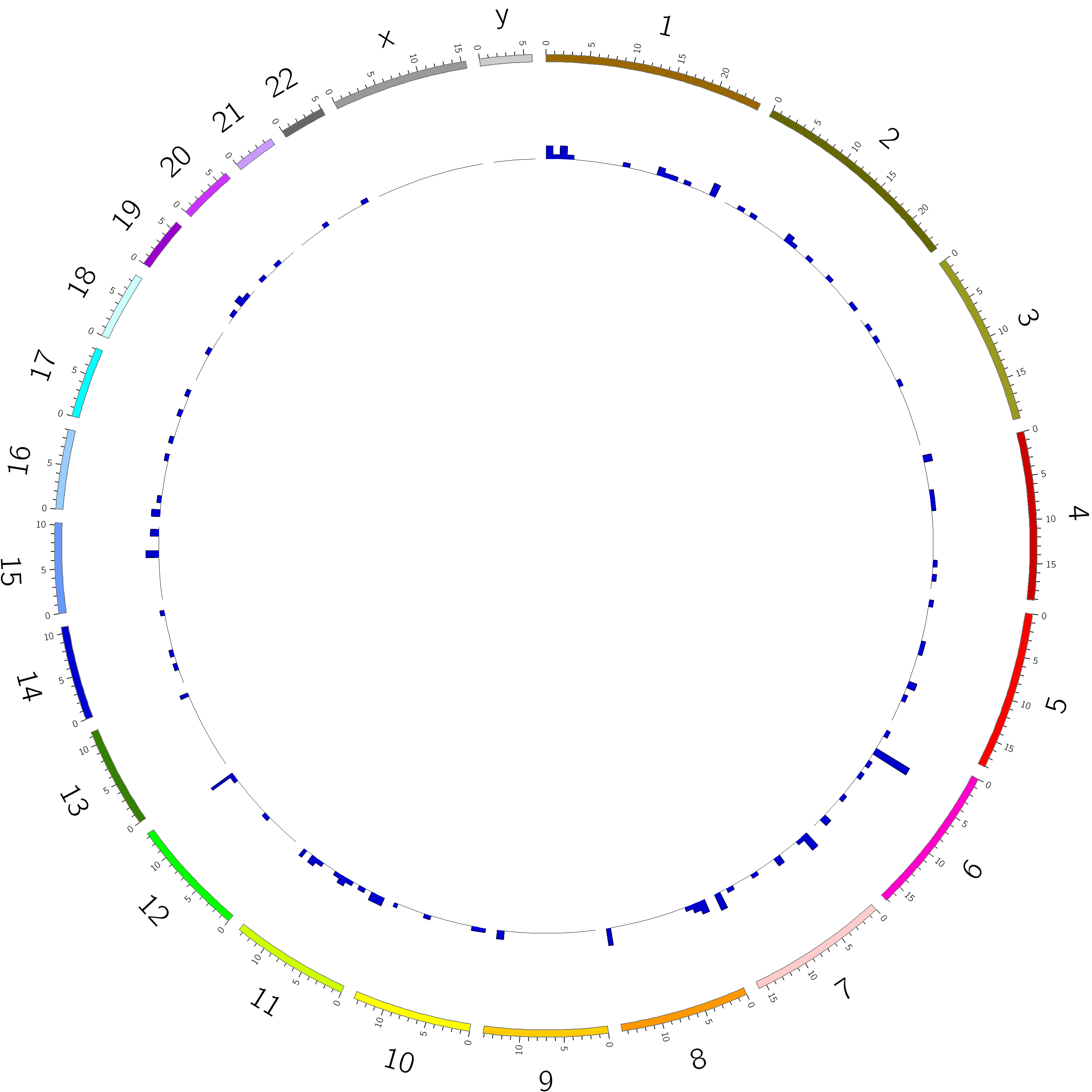
**

**eFigure 9. The distribution of differential methylation sites on chromosomes.** Taking 10mbp as a window, the number of different methylation sites in the window was counted, and the distribution of sites on chromosome was obtained. (a): The comparison between HMAQ group and LMAQ group; (b): The comparison between HMAQ group and Health control group; (c): The comparison between LMAQ group and Health control group.

**eFigure 10**

**
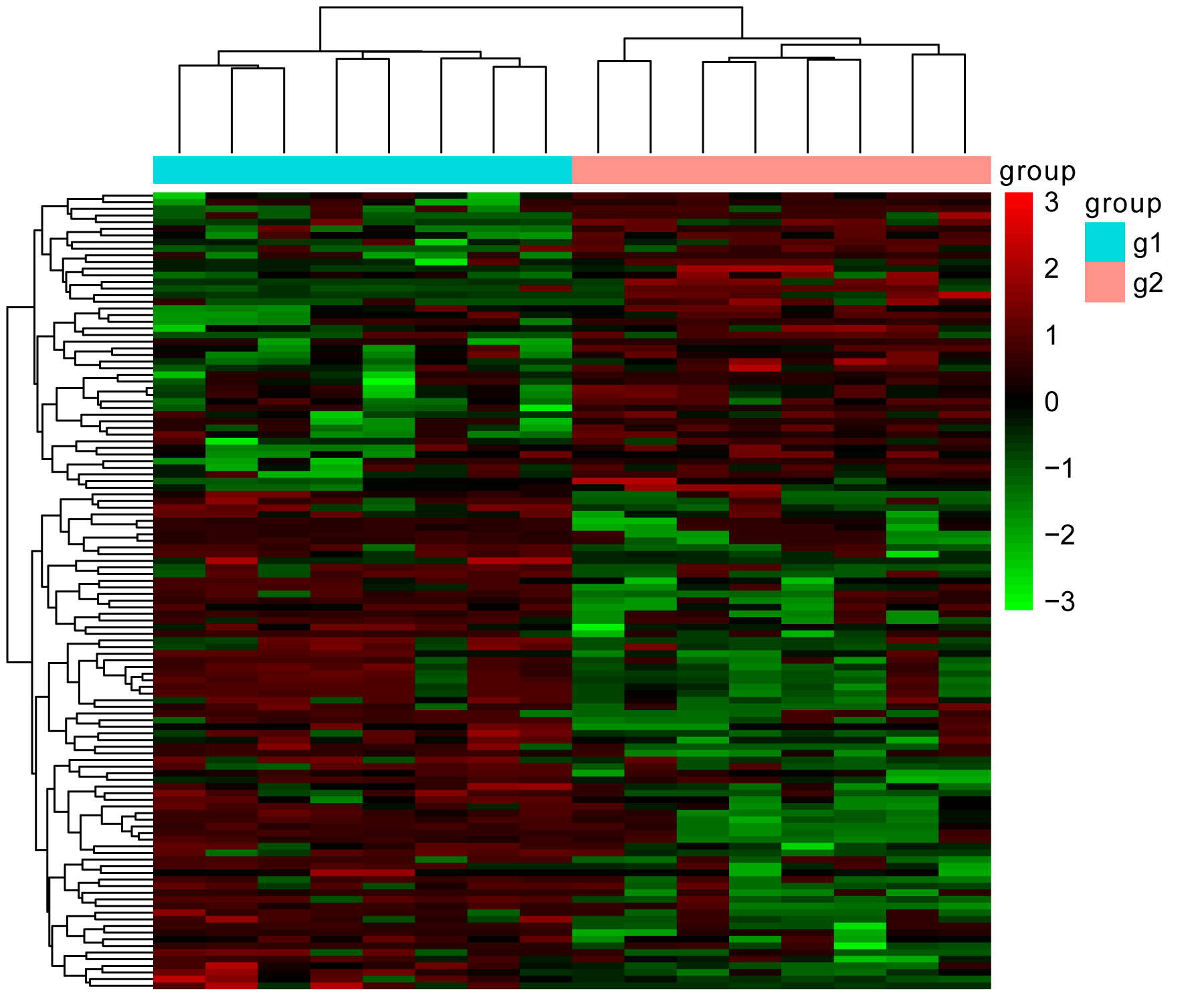
**

**
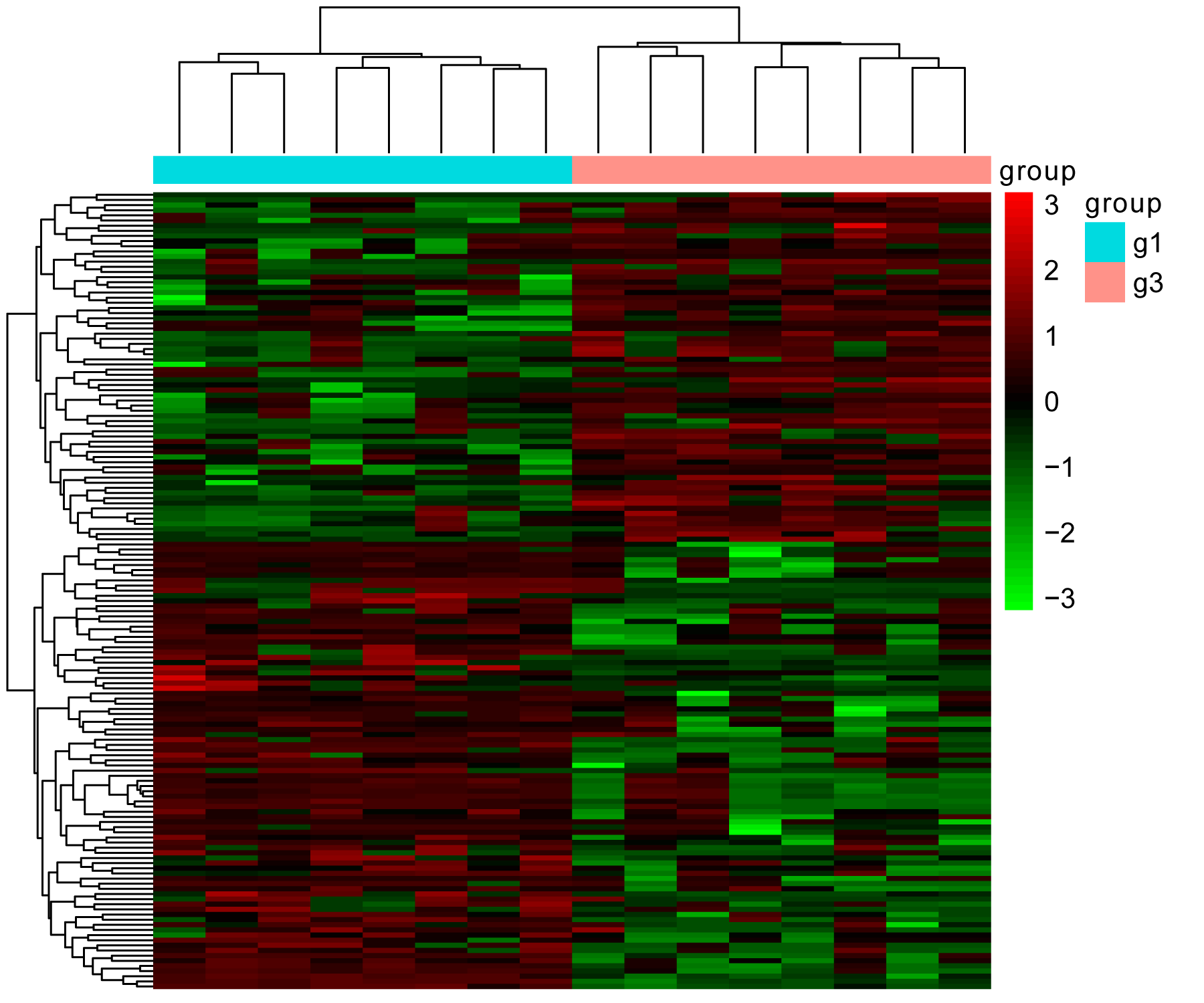
**

**
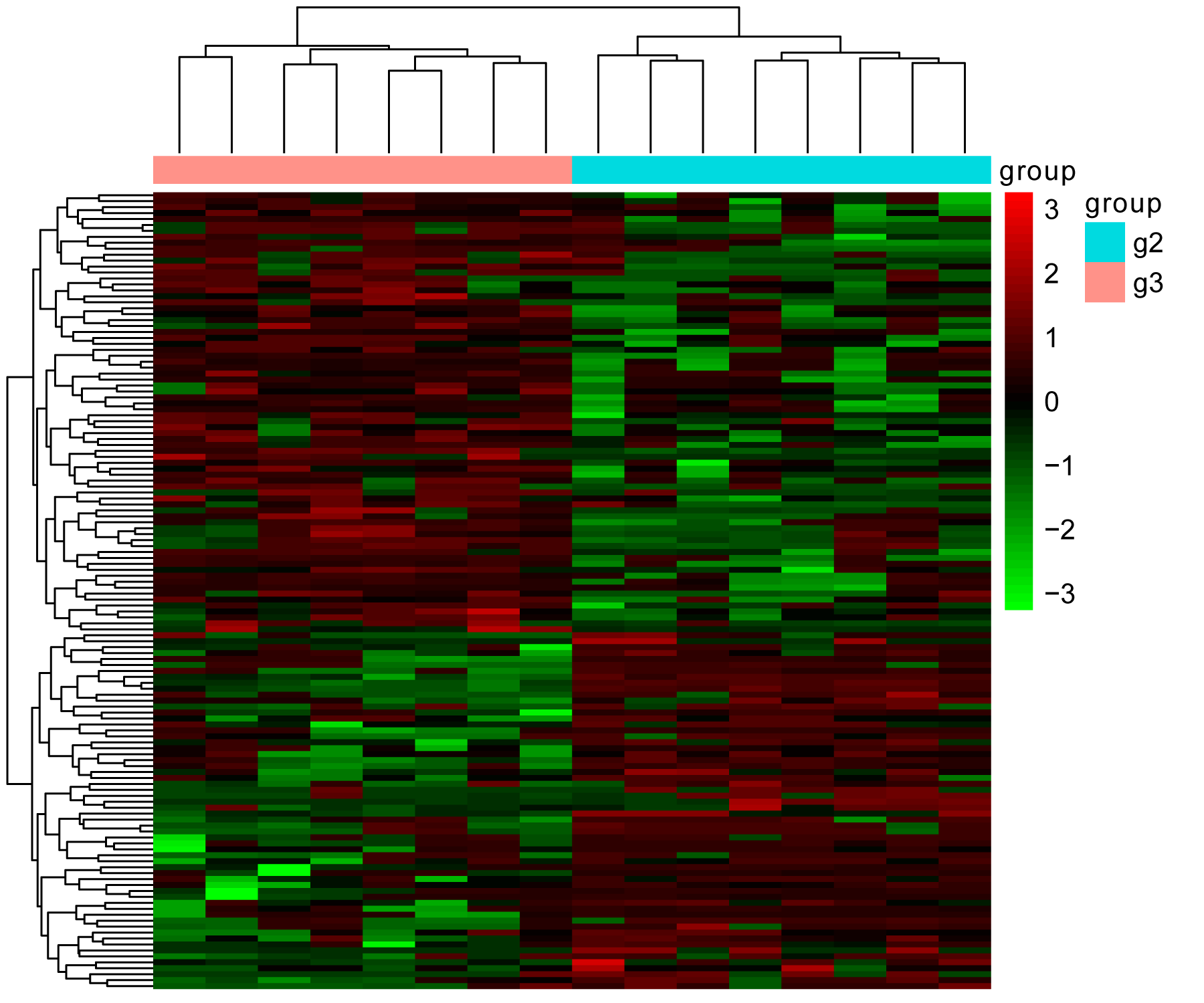
**

**eFigure 10. The heatmap of the differentials of methylation sites in pairwise comparisons of three groups.** The cluster analysis results showed the differentials of methylation sites between groups. Red means high methylation status, and green means low methylation status. The darker color means the more significant methylation differential. g1: HMAQ group; g2: LMAQ group; g3: Health control group.

**eFigure 11**

**
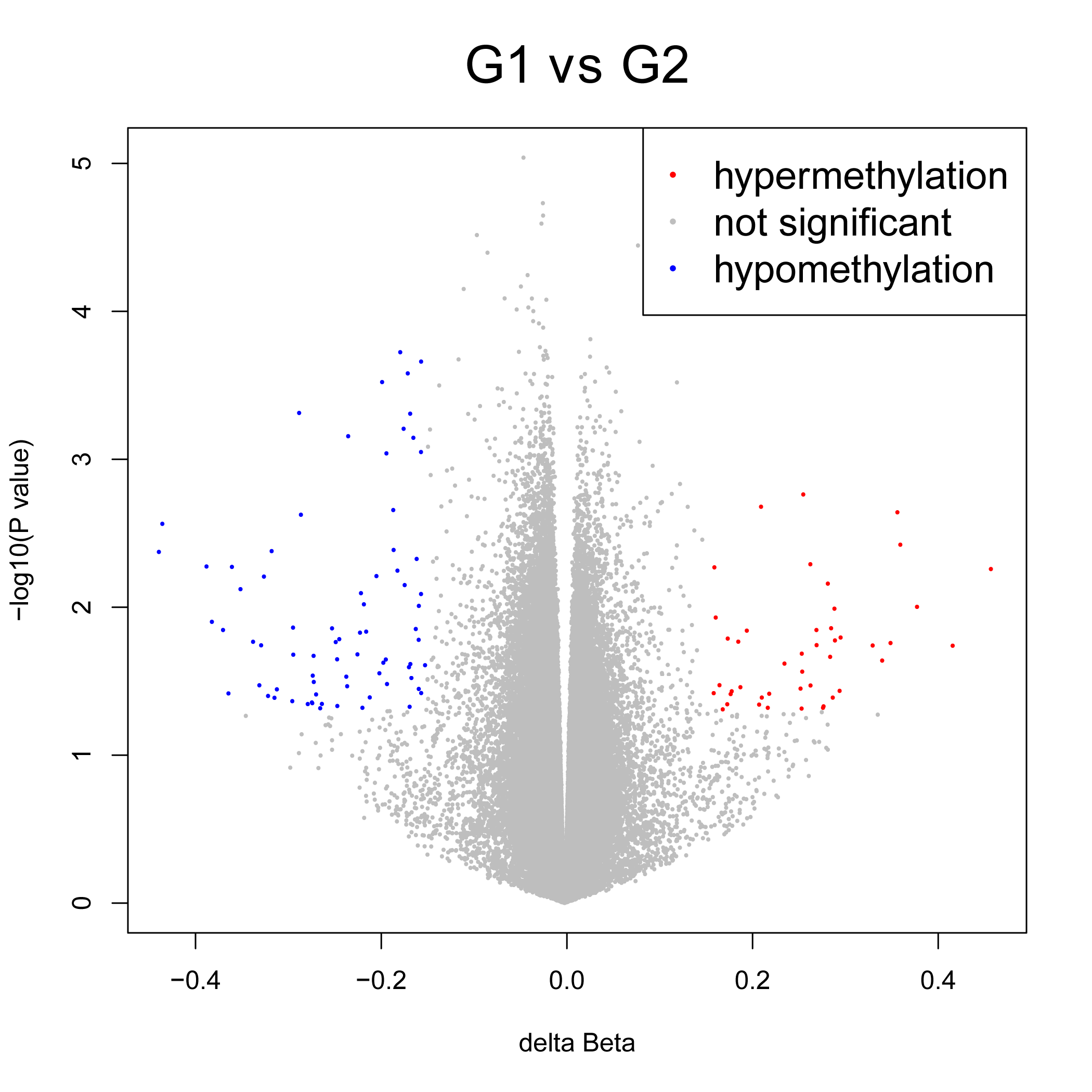
**

**
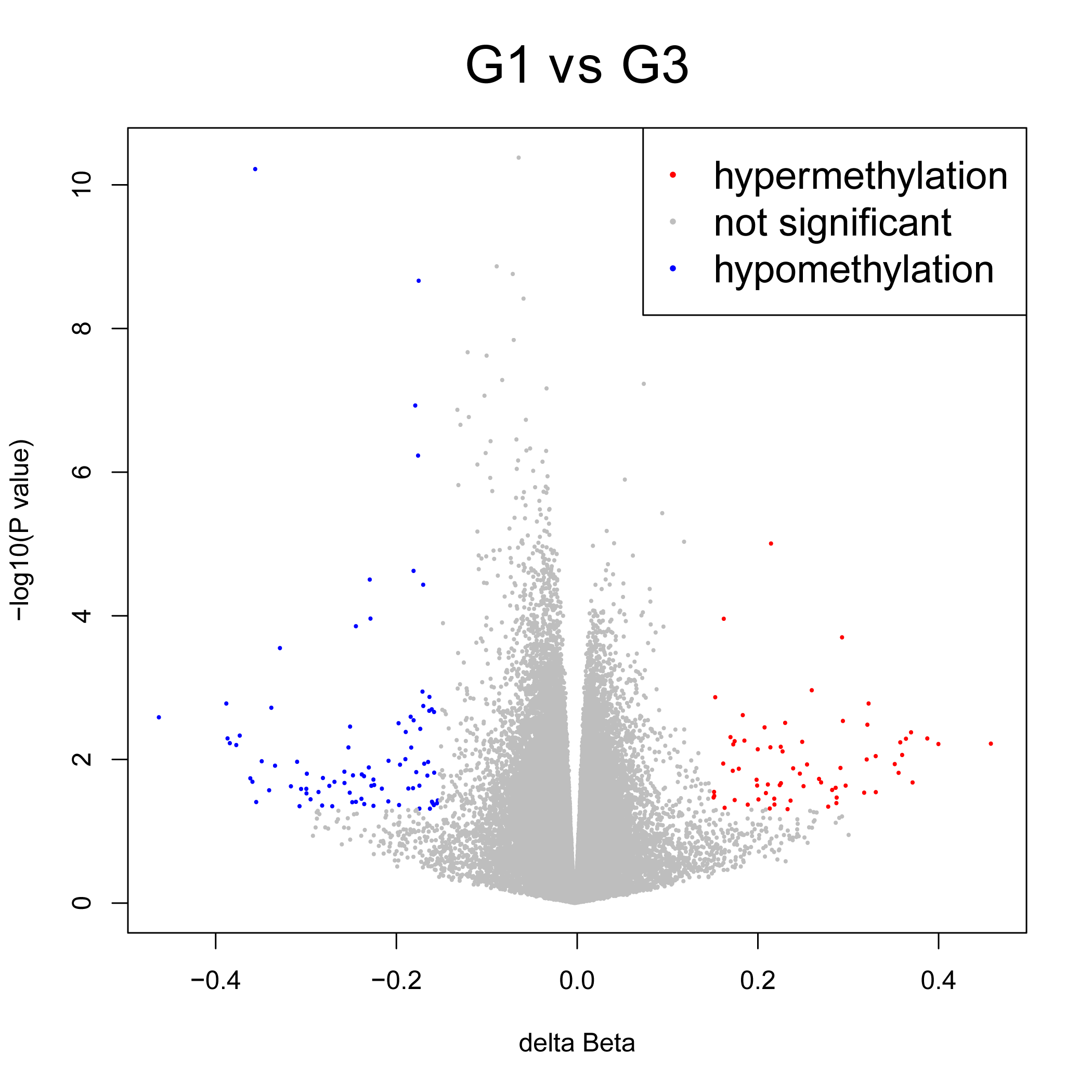
**

**
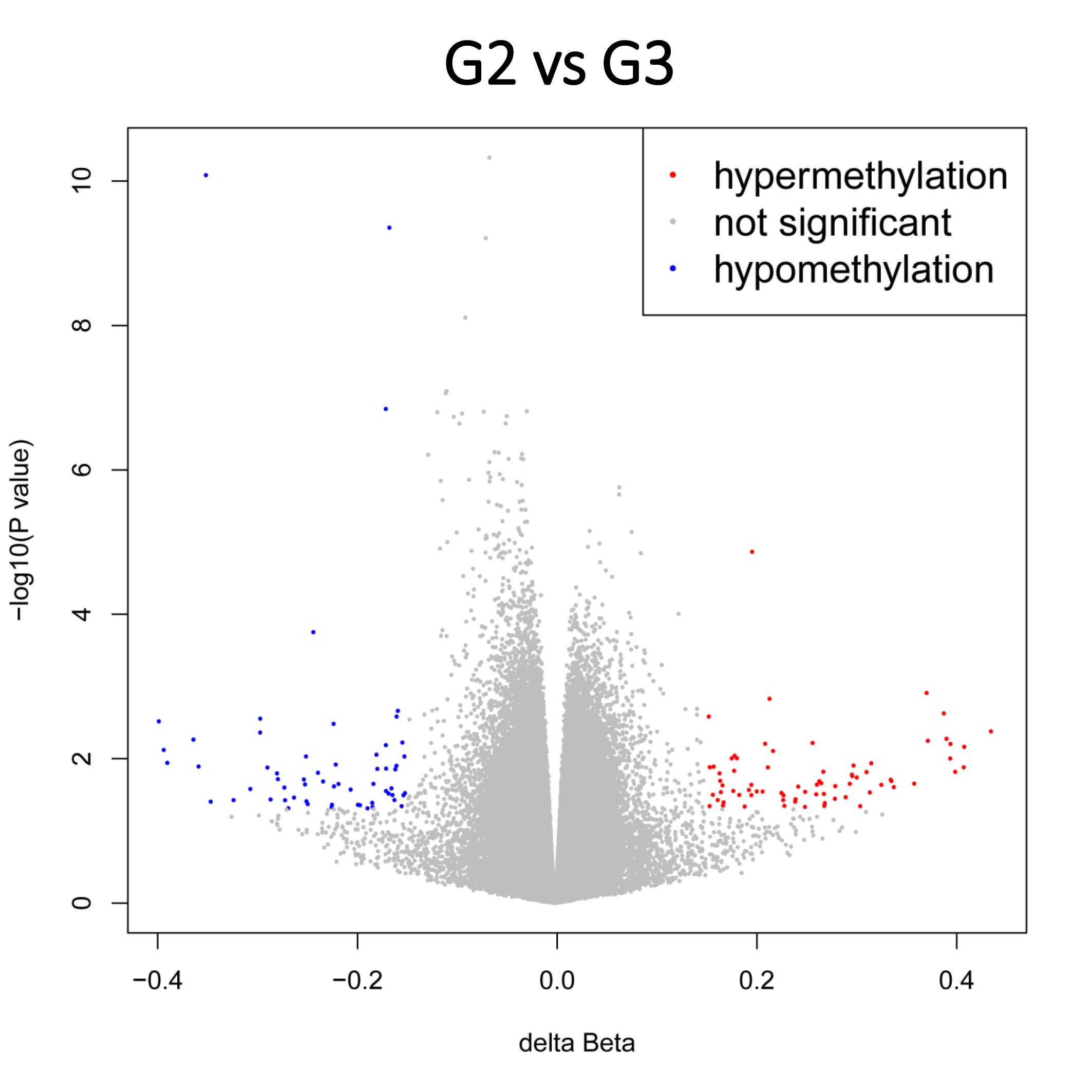
**

**eFigure 11. The volcano plot of differential methylation sites in pairwise comparisons of three groups.**

In the three volcano plots, the abscissa is delta Beta value, and the ordinate is -logpvalue. The blue rendering points are low methylation sites, and the red rendering sites are high methylation sites. G1: HMAQ group; G2: LMAQ group; G3: Health control group.
